# Diabetic Foot Ulceration in Dialysis‐Dependent End‐Stage Kidney Disease: A Systematic Review of Epidemiology, Clinical Outcomes and Mortality Risk

**DOI:** 10.1002/dmrr.70189

**Published:** 2026-06-16

**Authors:** J. Z. M. Lim, D. Selvarajah, S. Mitra, N. S. L. Ng, G. Rayman, L. Vileikyte, F. L. Game, A. J. M. Boulton

**Affiliations:** ^1^ Diabetes, Endocrinology and Metabolism Centre Manchester Royal Infirmary Manchester University NHS Foundation Trust Manchester UK; ^2^ Division of Diabetes, Endocrinology and Gastroenterology Faculty of Biology, Medicine and Health University of Manchester Manchester UK; ^3^ Department of Cardiovascular and Metabolic Medicine Institute of Life Course and Medical Sciences, University of Liverpool Liverpool UK; ^4^ Department of Oncology and Metabolism Faculty of Health University of Sheffield Sheffield UK; ^5^ Department of Nephrology Manchester University NHS Foundation Trust Manchester UK; ^6^ Manchester Academy of Health Sciences Centre (MAHSC) Manchester UK; ^7^ Department of Paediatric Nephrology Royal Manchester Children's Hospital Manchester UK; ^8^ Diabetes Centre Ipswich Hospital East Suffolk and North Essex NHS Foundation Trust Ipswich UK; ^9^ Faculty of Health and Medicine Lancaster Medical School Lancaster University Lancaster UK; ^10^ Department of Diabetes and Endocrinology University Hospitals of Derby and Burton NHS Foundation Trust Derby UK

**Keywords:** diabetic foot ulcer, dialysis, end‐stage kidney disease, lower‐extremity amputation, mortality, wound healing

## Abstract

**Background:**

Diabetic foot ulceration (DFU) and lower limb complications are highly prevalent in people with end‐stage kidney disease (ESKD), particularly those receiving dialysis; however, the overall burden and outcomes remain incompletely characterised. This systematic review with narrative synthesis aimed to summarise study characteristics and evidence on the epidemiology of DFU in ESKD, including incidence, prevalence, wound healing outcomes, and associations with lower‐extremity amputation (LEA) and mortality.

**Methods:**

MEDLINE (via PubMed), EMBASE and the Cochrane Database of Systematic Reviews were searched from inception to 31 January 2026 for longitudinal and cross‐sectional studies, including registry data, in adults with ESKD or on dialysis. Outcomes included DFU epidemiology, wound healing, revascularisation, LEA and mortality.

**Results:**

The review included 64 observational studies. In dialysis‐dependent populations, DFU incidence is high and increases with advancing renal impairment, often preceding dialysis initiation. Evidence on whether dialysis initiation itself increases DFU risk is limited and heterogeneous, although observational cohorts suggest a temporal association with haemodialysis initiation, particularly within the first 2 years. Data comparing haemodialysis and peritoneal dialysis are scarce. Wound healing outcomes were variable, with earlier recurrence observed, although multidisciplinary care improved healing, largely driven by perfusion and ulcer severity rather than renal function alone. Although based on observational and heterogeneous data, dialysis‐dependent ESKD was frequently identified as an independent predictor of LEA after adjustment for confounders, with coexisting peripheral arterial disease, a key determinant of adverse limb outcomes. Mortality risk appeared to compound following amputation, with observational data suggesting high post‐amputation mortality (approaching 50% at 2 years and 70% at 5 years), consistent with a shift towards limb loss rather than increased DFU occurrence. Interpretation is limited by study heterogeneity, observational design, limited long‐term data on healing and recurrence, and inadequate stratification by dialysis modality.

**Conclusions:**

Current evidence underscores substantial gaps in understanding the natural history and optimal management of diabetic foot disease in dialysis‐dependent ESKD. Future research should prioritise well‐designed prospective studies to delineate dialysis‐specific risk pathways, incorporate robust stratification by dialysis modality, and evaluate interventions targeting ischaemia and limb preservation. Standardisation of outcome reporting, particularly for healing durability and recurrence, will be essential to enable meaningful comparisons and guide the development of tailored multidisciplinary care models for this high‐risk population.

**Abbreviations**
CKD: Chronic Kidney Disease
CVD: Cardiovascular Disease
DFU: Diabetic Foot Ulcer
DM: Diabetes Mellitus
DOPPS: Dialysis Outcomes and Practice Patterns Study
DPN: Diabetic Peripheral Neuropathy
eGFR: estimated Glomerular Filtration Rate
ESKD: End‐Stage Kidney Disease
HD: Haemodialysis
HR: Hazard Ratio
IWGDF: International Working Group on the Diabetic Foot
LEA: Lower Extremity Amputation
MDT: Multidisciplinary Team
MeSH: medical subject headings
NDFA: National Diabetes Foot Care Audit
NIHR: National Institute for Health and Care Research
NR: not reported
OR: Odds Ratio
PAD: Peripheral Arterial Disease
PD: Peritoneal Dialysis
PRISMA: Preferred Reporting Items for Systematic Reviews and Meta‐Analyses
RCT: Randomised Controlled Trial
RRT: Renal Replacement Therapy
STROBE: Strengthening the Reporting of Observational Studies in Epidemiology
TcPO_2_
: Transcutaneous Oxygen Pressure
USRDS: United States Renal Data System
YHEC: York Health Economics Consortium

## 1 Introduction

Diabetes‐related foot ulceration (DFU) and chronic kidney disease (CKD) are major complications of diabetes, encompassing both microvascular and macrovascular pathology, and are each independently associated with a markedly increased risk of cardiovascular disease (CVD), the leading cause of death in this population [1, 2, 3]. Globally, among the more than half a billion individuals living with diabetes, approximately 19%–34% will develop DFU and up to 40% will develop CKD during their lifetime, with diabetes remaining the principal driver of the growing demand for haemodialysis (HD) and other forms of renal replacement therapy [2, 4, 5, 6]. DFUs are a major cause of morbidity and mortality [7] among people with diabetes, frequently leading to infection, hospitalisation, lower‐extremity amputation (LEA), and premature death [8].

While biological drivers such as peripheral arterial disease (PAD), peripheral neuropathy (DPN), and impaired wound healing are well‐established determinants of poor outcomes in DFU, psychosocial factors‐particularly depression ‐are increasingly recognised as critical but under‐appreciated contributors to prognosis. Depression affects around one‐third of individuals at first DFU presentation and is independently associated with markedly higher mortality [9], including a persistent two‐to three‐fold excess risk of mortality [10, 11] over 5 years. It is also linked to increased LEA and greater diabetes‐related distress [10], and may precede DFU development, with evidence of a dose‐response relationship between depressive symptom severity and incident DFU [12, 13]. Depression is likewise highly prevalent in HD populations and is associated with impaired quality of life [14] and independently predicts poorer survival [15]. Despite this overlap, outcomes in individuals with DFU, ESKD, and depression remain poorly characterised [10, 11, 15], highlighting that depression may amplify adverse outcomes in dialysis populations [14].

Impaired healing of DFU is driven by microcirculatory dysfunction superimposed on an already high‐risk vascular profile. HD introduces repeated haemodynamic stresses that directly reduce tissue oxygenation. Transcutaneous oxygen pressure (TcPO_2_) studies [16] show that during HD, severe limb ischaemia occurs in nearly half of patients and critical ischaemia in a substantial minority, often linked to intradialytic hypotension, ultrafiltration‐related volume shifts, and underlying PAD. Because hypotension occurs in up to half of dialysis sessions [17], recurrent reductions in oxygen delivery are common and biologically plausible contributors to impaired fibroblast activity, reduced angiogenesis, and delayed inflammatory resolution [18]. PAD is highly prevalent in CKD and ESKD, where arterial calcification, stiffness, and poor collateralisation limit compensatory perfusion, leaving tissues vulnerable to these dialysis‐related insults. Coexisting DPN, affecting most dialysis patients, further increases risk through loss of protective sensation, abnormal biomechanics, and repetitive pressure injury. Inconsistent foot surveillance within dialysis pathways may also delay recognition and referral [19]. Together, these vascular [20], neurological, and system‐level factors create a persistently hypo‐perfused [21] wound environment that contributes to poor DFU healing and high limb‐loss rates [22]. Patient and public involvement work has emphasised the need for therapies that are both effective and feasible within dialysis care [23]. The aim of this systematic review is to synthesise existing evidence from studies quantifying the epidemiology and disease burden of DFU in adults receiving HD, examine associations with LEA and mortality, and identify patient populations at greatest risk.

## 2 Methodology

### 2.1 Search Strategy

**FIGURE 1:**
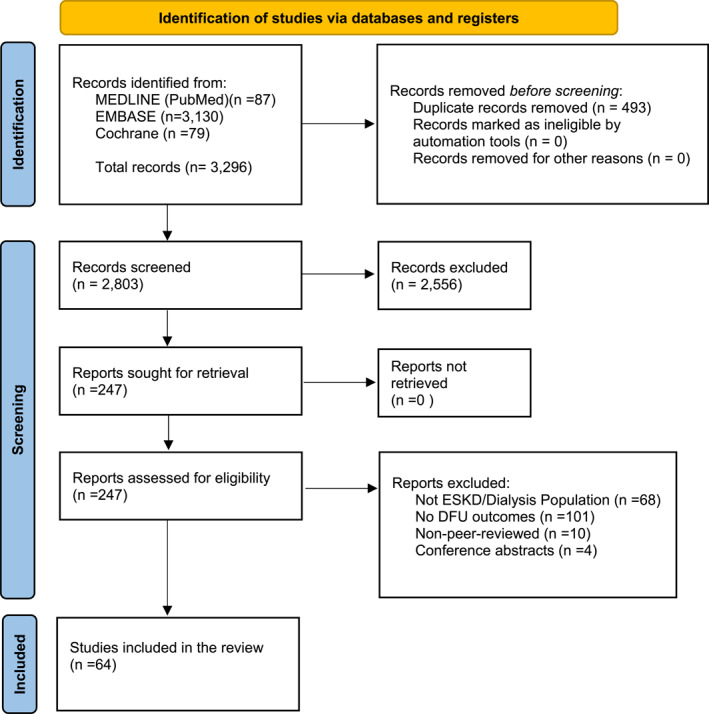
PRISMA flow diagram of study selection.

A systematic literature search was conducted from database inception to 31 January 2026 using MEDLINE (via PubMed), EMBASE and the Cochrane Database of Systemic Reviews. Searches were restricted to English‐language publications. The search strategy combined keywords and medical subject headings (MeSH) related to DFU, DPN, PAD, CKD, ESKD, renal replacement therapy, and HD. Full electronic database search strategies are provided in Supporting Information S1: Appendix 1: Search Strategies. The search strategy identified 3296 records (MEDLINE 87, EMBASE 3,130, Cochrane 79). After removal of 493 duplicates, 2803 records remained. Following preliminary screening, 2556 records were excluded, leaving 247 articles for full‐text review. Of these, 183 were subsequently excluded—most commonly due to non‐dialysis populations or absence of DFU‐specific outcomes—resulting in 64 studies included in the final synthesis. The PRISMA flow diagram is shown in Figure 1. This review was not prospectively registered. A formal review protocol was not prepared; however, predefined inclusion and exclusion criteria, a PICO framework, and a structured search strategy were developed and followed throughout the review. The full search strategy is available in Supporting Information S1: Appendix 1: Search Strategy.

### 2.2 Study Selection

Titles, abstracts, and full texts were screened against predefined inclusion and exclusion criteria. Full‐text screening and data extraction were performed independently by two reviewers (JL and DS), with study eligibility for each synthesis determined through comparison of extracted data against predefined criteria. Disagreements were resolved by discussion and, where necessary, by consensus with a third reviewer (NN).

Eligible studies included observational designs (prospective and retrospective cohort studies and cross‐sectional analyses), registry‐based studies, and systematic reviews evaluating DFU and/or LEA in adults with ESKD receiving dialysis. Conference abstracts, unpublished studies, and non–peer‐reviewed reports were excluded.

### 2.3 Data Extraction and Quality Assessment

Data extracted from each study included study design, country and clinical setting, population characteristics, dialysis modality, proportion of participants with diabetes, study objectives, comparison groups, follow‐up duration, and reported outcomes. Effect estimates (e.g., odd ratios, hazard ratios, risk ratios) and variables adjusted for in multivariable analyses were also recorded. Risk of bias was assessed using STROBE criteria, evaluating participant selection, exposure and outcome definitions, statistical methods, and adjustment for confounding. Detailed quality assessment is presented in Supporting Information S3: Appendix 3 (STROBE checklist of included studies), with the scoring framework provided in Table S1.

### 2.4 Data Synthesis and Identification of Evidence Gaps

Study identification and data extraction were conducted systematically. Given the predominantly observational nature of the included studies and substantial clinical and methodological heterogeneity—particularly in patient characteristics (age, diabetes and dialysis duration, baseline foot risk), outcome definitions, and analytical approaches—quantitative synthesis was not appropriate. Definitions of DFU, lower extremity amputation, and follow‐up duration varied considerably, and effect estimates were reported using different measures and adjustment models, limiting comparability. Key exposures such as PAD, diabetic peripheral neuropathy, CKD severity, and prior DFU were also inconsistently defined. Findings were synthesised narratively to describe patterns in DFU epidemiology, wound healing, LEA and mortality in ESKD or dialysis populations.

### 2.5 Study Characteristics

Across the 64 included studies, the mean STROBE score was 16.9, indicating an overall moderate reporting quality. Most studies were of moderate quality (*n* = 46), with fewer classified as high quality (*n* = 16) and only two as low quality (see Supporting Information S3: Appendix 3: STROBE Checklist). All included studies were observational, published between 1992 and 2026, and conducted across multiple geographic settings; detailed study characteristics are presented in Table 1. Most were hospital‐ or specialist clinic‐based, with follow‐up ranging from weeks to > 10 years, commonly reporting 1‐ and 5‐year mortality. Sample sizes varied widely, from small single‐centre cohorts to large registry datasets (> 100,000 patients) [24, 25, 26, 27, 28], with substantial variation in dialysis representation. Across studies (*n* = 559,800), 30.4% were on HD and 0.2% on PD, with diabetes present in most cohorts. LEA (*n* = 41) and mortality (*n* = 35) were most frequently reported, while incidence, prevalence, and healing outcomes were less commonly described. Over half of the included studies (31/59; 52.5%) comprised cohorts in which dialysis patients represented less than 75% of the study population, reflecting substantial heterogeneity in population composition.

**TABLE 1:** Characteristics of included studies evaluating diabetic foot ulceration in dialysis‐dependent ESKD.

General	*N*	%
Experimental or observational		
Observational	64	100
Study design		
Cohort study	55	85
Case series	1	1
Cross sectional	8	14
Clinical setting of intervention		
Dialysis unit	32	52
Outpatient setting (other than dialysis)	12	18
National data registry	6	10
Inpatient (hospital setting) (other than dialysis)	13	20
Country		
United States of America	13	20
United Kingdom	6	9
Spain	5	8
France	3	5
Germany	3	5
Italy	6	9
Netherlands	2	3
Belgium	1	1.5
Finland	1	1.5
Czech Republic	1	1.5
Australia	4	6
China	2	3
South Korea	5	8
Japan	5	8
Taiwan	1	1.5
Singapore	1	1.5
Kuwait	1	1.5
Qatar	1	1.5
Argentina	1	1.5
Nigeria	1	1.5
Iran	1	1.5
Libya	1	1.5
Multiple countries (European and North America)	3	5
Duration of follow‐up		
Less than 6 months	9	14
6 months to less than 1 year	12	19
1 year to less than 2 years	22	34
2 years to less than 5 years	9	14
5 years or more	12	19
Population		
Proportion of dialysis versus non‐dialysis patients included in the study		
< 75%	34	53
≥ 75%	30	47
Type of dialysis		
Haemodialysis	28	44
Haemodialysis and peritoneal dialysis	10	16
ESKD or CKD5	26	40
Diabetes		
Yes	64	100
No	0	0

## 3 Results

### 3.1 Epidemiology of DFU Across CKD Progression

Up to 40% of individuals with diabetes are estimated to develop CKD [29]. Across diverse study designs and healthcare systems, diabetic foot disease demonstrates a strong and consistent association with declining renal function [30, 31]. Epidemiological data show a graded increase in both the prevalence [32] and incidence of DFU from moderate CKD through to ESKD, with the highest burden observed in people receiving dialysis [33]. Large cohort and registry studies report that individuals with diabetes and ESKD account for a disproportionately high share of diabetic foot complications, including ulceration, amputation [34], and foot‐related hospitalisation, compared with people with preserved renal function. DFU represents one of the most severe and disabling complications of diabetes and is markedly over‐represented in people with advanced CKD and ESKD [35].

Observational data demonstrate a graded relationship between declining renal function and increasing podiatric risk. Mean eGFR decreased across IWGDF risk stages, from 76.4 ± 33.7 mL/min/1.73 m^2^ in stage 0–48.2 ± 30.1 mL/min/1.73 m^2^ in stage 2, with significant differences between stages (*p* ≤ 0.02). Consistent with this finding, the prevalence of higher podiatric risk (IWGDF stage ≥ 2) [36] increased significantly with worsening renal function (*p* = 0.0001), highlighting advanced CKD as a key determinant of diabetic foot disease burden [31].

### 3.2 Incidence of DFU in ESKD on Dialysis Population

Evidence on the incidence of DFU in dialysis populations remains limited, but available studies consistently demonstrate a substantial and early burden of disease [31, 33, 34, 35, 37, 38, 39, 40, 41, 42, 43, 44, 45, 46, 47, 48], with study characteristics and incidence data summarised in Table 2, and the complete extracted study characteristics and outcome dataset presented in Supporting Information S2: Appendix 2. In a prospective cohort of 450 patients receiving dialysis (94% HD; 6% PD), with a high prevalence of diabetes (50%), PAD (34%), and DPN (65%), the annual incidence of DFU was 18% [37]. The annual incidence rate was estimated at 122 per 1000 person‐years [37], markedly higher than rates reported in general diabetes populations [47]. Within the cohort study, DFU occurred early, with a mean time to onset of 164 days, highlighting rapid progression to complications in this high‐risk group [37].

**TABLE 2:** Characteristics of studies reporting the prevalence and incidence of DFU in patients with ESKD receiving dialysis.

Study (author, year)	Country/setting	Study design	Data source	Study period	Population, dialysis & vascular risk profile	Comparator group	DFU prevalence (%)	DFU incidence
Kaminski et al. [37]	Australia	Prospective cohort	Dialysis units + clinical exam	12 months	*N* = 450; HD 94%, PD 6%; diabetes 50%; PAD 34%; DPN 65%	DFU versus no DFU	21.6% (prior DFU)	18% at 12 months; 122/1000 PY
Al‐Thani et al. [40]	Qatar	Retrospective cohort	HD clinical records	5 years	*N* = 252 HD; high PAD/CVD burden	FU versus no FU	23%	NR
Game et al. [38]	UK	Case‐series	RRT + foot clinic databases	1976–2006	*N* = 466 RRT; diabetes 100%	Pre versus post dialysis	20.2%	IR 3.35–4.56 post‐RRT
Lavery et al. [42]	USA	Retrospective cohort	Claims + EMR	Pre/post dialysis	*N* = 150; T2DM dialysis	Pre versus post dialysis	NR	91.7 versus 82.7/1000 PY (pre vs. post)
Otte et al. [34]	Netherlands	Retrospective cohort	Hospital records	2006–2012	*N* = 669; dialysis *n* = 259; PAD 26.6%	CKD stage comparison	NR	104/1000 PY (dialysis); HR 7.6
Ndip et al. [33]	UK	Cross‐sectional	Diabetes/renal clinics	NR	*N* = 326; CKD4–5; diabetes 100%; PAD 64%	Dialysis versus non‐dialysis	21% versus 5%	NR
Ndip et al. [33]	UK/USA	Cross‐sectional	Dialysis clinics	NR	*N* = 466; HD predominant; PAD 57%	None	12%	NR
Dòria et al. [44]	Spain	Retrospective cohort	Dialysis records	5–6 years	*N* = 220 HD; diabetes 38.6%	Diabetes versus no diabetes	9.1% active	↑ cumulative DFU/amputation risk
Dòria et al. [41]	Spain	Cross‐sectional	Dialysis assessment	NR	*N* = 92 HD/PD; PAD 64%	None	17.4%	NR
Spinelli et al. [43]	Argentina	Cross‐sectional	Hospital records	2022–2024	*N* = 54; HD 73%, PD 27%; PAD 81.5%	None	40.7%	NR
Holman et al. [27]	UK	Prospective cohort	NDFA registry	2017–2022	*N* = 71,000; HD subgroup 3.4%	RRT versus non‐RRT	3.4% (HD subgroup)	NR
Felipe et al. [45]	Spain	Cross‐sectional	Hospital cohort	2017–2018	*N* = 104; 73% dialysis; high PAD/DPN	None	6%	NR
Hernandez et al. [49]	Spain	Retrospective cohort	Dialysis cohort	7 years	*N* = 202; 94% HD; PAD 31%	None	29.7% (49% in DM)	NR
Ozdemir et al. [50]	Turkey	Cross‐sectional	Clinical assessment	2017	*N* = 180 HD; high neuropathy	DFU versus no DFU	6.7% active; 19.4% history	NR

Abbreviations: HD = haemodialysis; IR = incident rate; NR = not reported; PD = peritoneal dialysis; RRT = renal replacement therapy.

The risk of DFU is already elevated prior to dialysis initiation and remains high thereafter [42]. In one cohort, the incidence of first DFU was similar before and after starting haemodialysis (91.7 vs. 82.7 per 1000 patient‐years), indicating that substantial risk precedes renal replacement therapy [42]. When recurrent events were included, cumulative incidence exceeded 200 per 1000 patient‐years [42], with many patients experiencing multiple DFU episodes. Although the cumulative burden of ulceration was higher prior to dialysis, the incidence of first DFU remained consistently high before and after initiation, indicating that patients with advanced CKD and ESKD already carry a persistently elevated risk of ulceration [42].

This gradient of risk is further supported by a large retrospective study demonstrating a stepwise increase in DFU incidence across CKD stages, rising from 12 per 1000 patient‐years in CKD stage 3–47 in stage 4–5 and 104 in dialysis patients [34]. After adjustment, dialysis conferred a 7.6‐fold higher risk of DFU than CKD stage 3 [34]. Cross‐sectional data from UK cohorts reinforce this finding, showing a higher prevalence of active DFU in HD versus non‐HD CKD stage 4–5 patients (21% vs. 5%), alongside a high burden of DPN (79%) and PAD (64%), with HD identified as an independent risk factor [33].

Temporal analyses further highlight a critical period of vulnerability around dialysis initiation. In a UK self‐controlled case‐series, 20.2% of patients developed DFU [38], with incidence increasing more than three‐fold in the first year of dialysis (incidence ratio 3.35) and remaining over four‐fold higher in years two to five (incidence ratio 4.56) [38]. These findings suggest sustained excess risk following transition to renal replacement therapy [38].

Prevalence data from dialysis cohorts similarly demonstrate a high and variable burden of DFU. Reported prevalence ranges from 9.1% in HD cohorts [44] to 17%–23% in populations with high vascular risk [40, 41], and up to 40.7% in cohorts enriched for critical limb ischaemia [43]. Even where active DFU prevalence appears lower (e.g., 6% in mixed CKD stage V cohorts) [45], a substantial proportion of patients (up to 49%) exhibit broader foot pathology, indicating a large at‐risk population [31]. Across studies, high rates of neuropathy and PAD consistently underpin this burden.

In contrast, the annual incidence of DFU in the general diabetes population is typically 1.9%–4% [46, 47, 48], rising modestly in those with neuropathy [8]. By comparison, dialysis populations frequently demonstrate annual incidence rates approaching or exceeding 10%–20% [37, 42], with cumulative rates surpassing 200 per 1000 person‐years in some cohorts [42]. Although population‐level surveillance data in dialysis remain limited, the consistency and magnitude of these findings, together with evidence of temporal clustering around dialysis initiation [38], support dialysis as a major independent modifier of DFU risk [33] rather than simply a marker of longstanding diabetes.

### 3.3 Prevalence of DFU in ESKD on Dialysis Population

The prevalence of DFU in patients with ESKD receiving HD is consistently higher than in non‐dialysis diabetes populations. Cohort studies consisting of patients with diabetes and ESKD on HD demonstrate substantially elevated prevalence of DFU, typically ranging from 9.1% [44] to 23%, including 9.1% in a Spanish cohort [44], 17.4% in a diabetes‐only HD population [41], and 23% in a high vascular‐risk cohort [40]. A UK cross‐sectional study of adults with diabetes and advanced CKD (stages 4–5) attending tertiary diabetes, renal, and dialysis services demonstrated a high burden of foot disease [33]. The point prevalence of active DFU was 11%, with dialysis‐treated patients exhibiting a fivefold higher prevalence than non‐dialysis patients with comparable renal impairment [33]. Dialysis was associated with a higher prevalence of prior DFU, neuropathy and PAD, indicating clustering of foot‐specific risk factors in advanced CKD populations [33].

Supporting this, a large retrospective dialysis cohort reported that 20.2% of patients with diabetes developed at least one DFU, alongside a marked increase in ulcer incidence after dialysis initiation [38]. Similar patterns have been reported internationally; in a tertiary Argentinian cohort of patients with diabetes receiving renal replacement therapy, over 40% had diabetic foot disease, with active ulcers in 9.3% and pre‐ulcerative lesions in more than 60%, occurring alongside very high rates of DPN (87%), PAD (82%), and prior LEA in nearly one‐third of patients [43].

In a UK audit of HD patients, 79% had foot pathology despite DFU prevalence not being explicitly reported, with a high prevalence of PAD (37%) and DPN, with almost half exhibiting ≥ 2 major risk factors [51] highlighting a substantial burden of pre‐ulcerative disease. Similarly, in a retrospective cohort of 252 HD patients, DFU prevalence was 17%, with PAD emerging as the strongest independent predictor (adjusted OR 16.0) and contributing to markedly worse outcomes, including higher major amputation rates (26% vs. 1%) [40]. These findings are consistent across international cohorts, where clustering of PAD, DPN, and cardiovascular comorbidities underpins the high DFU risk observed in ESKD in HD populations [44, 52].

This high prevalence is compounded by the chronic and recurrent nature of DFU in advanced CKD and ESKD, with up to 69%–70% of patients experiencing recurrence over long‐term follow‐up and approximately 25%–28% recurring within the first year [53] and recurrence rates of ∼70% by 10–15 years [53]. Renal replacement therapy was associated with a significantly shorter time to recurrence (HR 3.71) [53], reflecting sustained vulnerability in this population. In parallel, national audit data demonstrate suboptimal healing, with only 43.4% of patients alive and ulcer‐free at 12 weeks, indicating that delayed healing further contributes to prolonged disease course and high recurrence rates [54].

### 3.4 Impact of Dialysis Modality (Haemodialysis vs. Peritoneal Dialysis)

UK audit data highlight important gaps in care delivery on dialysis units, with only 77% of dialysis patients receiving annual foot assessments, and limited representation of the PD populations [55]. Available data suggest suboptimal glycaemic control, with two‐thirds receiving therapies associated with hypoglycaemia and frequent hypoglycaemic episodes [55] in PD patients, although DFU‐specific outcomes remain under‐reported.

Observational evidence indicates that DFU risk is established prior to dialysis and persists following initiation of renal replacement therapy [38]. The incidence of DFU may increase after dialysis initiation in both HD and PD [38], with differing temporal patterns: in HD, the incidence of DFU is highest in the first year (incidence ratio 4.57), and declines over the subsequent 2–5 years, whereas in PD, a later peak is observed, reaching an incidence ratio of 6.78 during years 2–5 [38].

In a cohort of 14,935 Medicare beneficiaries with diabetes and ESKD on dialysis (HD 92.5%; PD 7.4%) who developed DFU, outcomes were poor, with 1‐year mortality of 44.8% and major amputation rates of 28.3%, yet only 18.4% received podiatry care in the preceding 3 months [26]. Direct comparisons between HD and PD are limited; one cross‐sectional study reported higher diabetic foot disease prevalence in HD versus PD (49% vs. 26%), with greater neuropathy, PAD, and prior amputation, although risk‐adjusted analyses are lacking [43]. Smaller cohorts (*n* = 466; 88% HD, 12% PD) similarly provide insufficient power for modality comparisons [33]. Cohort and population‐based data indicate that CKD and dialysis are major drivers of limb loss, with PD associated with a > 2.5‐fold increased risk of early major amputation (HR 2.56) and CKD nearly tripling risk (HR 2.97) [56], although evidence suggesting higher amputation risk in PD compared with HD is limited and derived from small, heterogeneous cohorts, precluding firm conclusions.

### 3.5 Risk Drivers of DFU in ESKD

Current evidence on DFU in ESKD is largely derived from HD populations, with limited mechanistic data. Renal dysfunction is consistently associated with increased DFU, LEA, and mortality, with particularly poor outcomes following amputation. The heightened risk in HD reflects the convergence of DPN, PAD, and advanced metabolic disturbance. Renal‐specific factors—including proteinuria, lower limb oedema, chronic inflammation, and malnutrition [57, 58]—further impair tissue integrity and wound healing, creating a hostile environment for wound repair.

PAD is highly prevalent in CKD and ESKD and a key driver of limb loss, characterised by diffuse distal disease with vascular calcification and impaired collateral formation, complicating both detection and revascularisation [59]. While PAD affects approximately 4% of the general population [59], its prevalence rises to 10%–34% in individuals with CKD and increases further with declining renal function [31, 60]. Ischaemia resulting from peripheral arterial occlusive disease is more prevalent and typically characterised by diffuse multi‐vessel involvement with a predilection for infra‐poplitaeal arteries [61, 62]. Neuropathy is similarly ubiquitous, affecting most dialysis patients, and contributes to sensory loss, altered biomechanics, and increased plantar pressure [43, 63, 64, 65]. Together with frailty, foot deformity, and delayed presentation [39, 40], these systemic and local factors underpin the high burden of DFU and poor limb outcomes in this population [66, 67].

### 3.6 Poor Healing Despite Revascularisation

The impact of ESKD and dialysis on DFU healing appears heterogeneous across the available literature, with healing and recurrence outcomes summarised in Table 3. While some studies report eventual long‐term healing, recurrence and adverse downstream outcomes remain consistently worse in this population. In long‐term European cohort data [53], healing rates were approximately 25%–28% at 1 year, increasing to 68%–70% over 10–15 years; however, ESKD was associated with significantly earlier ulcer recurrence (HR 3.71) [53], highlighting impaired durability of healing. In contrast, Tarricone et al. [69] demonstrated no significant differences in healing rates (63.0% vs. 58.1% vs. 58.1%), time to healing (106 vs. 155 vs. 116 days), re‐infection, or amputation across eGFR strata, suggesting a neutral effect of renal function on short‐term wound closure [69]; nevertheless, worsening CKD was associated with higher mortality and markedly increased rehospitalisation rates (up to 48.4%) [69]. Data from European MDT foot services [80], show overall favourable healing rates (67% at 12 months) despite a dialysis prevalence of 13%, with healing strongly influenced by perfusion and DFU burden rather than CKD status alone. Temporal cohort comparisons [72] further suggest that healing outcomes have improved over time despite increasing ESRD prevalence [25], likely reflecting advances in multidisciplinary care. Collectively, these findings indicate that while ESKD may not consistently impair initial DFU wound healing rates, it is associated with earlier recurrence [53], greater healthcare utilisation, and worse survival [53], underscoring the importance of long‐term risk rather than short‐term healing alone.

**TABLE 3:** Healing and recurrence outcomes in DFU with ESKD/dialysis.

Study (author, year)	Country/setting	Study design	Follow‐up duration	Population (N)	Dialysis/CKD subgroup	Healing outcomes	Recurrence
Ogurtsova et al. [68]	Europe (Germany, Czech Republic)	Prospective cohort	Up to 15 years	*n* = 321 (222 GER; 99 CZ)	ESKD on RRT subgroup (size NR)	NR	25%–28% (1 year); ∼68–70% (10–15 years); ESKD → earlier recurrence (HR 3.71)
Tarricone et al. [69]	USA, hospital cohort	Retrospective cohort	1 year	*n* = 344 (eGFR ≥ 60: *n* = 219; 30–60: *n* = 63; < 30: *n* = 62)	CKD stratified; eGFR < 30 representing advanced CKD/ESKD; higher PAD (up to 87.1%) and inflammation in low eGFR group	No difference by eGFR; healing: 63.0% versus 58.1% versus 58.1% (*p* = 0.70); time to heal: 106 versus 155 versus 116 days (*p* = 0.30)	Similar reinfection; ↑ rehospitalisation with CKD severity
Oh et al. [70]	South Korea; surgical cohort	Retrospective cohort	Mean 53.2 months	*n* = 113 (121 reconstructions)	Renal failure/transplant subgroup (size NR)	Healing 91.7%	NR
Honda et al. [71]	Japan; EVT centres	Observational cohort	Median 390–449 days	*n* = 267 (341 limbs; 380 wounds)	HD *n* = 120 versus non‐HD *n* = 147	Healing: 79.5% (HD) versus 92.4% (non‐HD), *p* < 0.001; HD RR 0.46	57.9% versus 35.9%, *p* = 0.004; HD RR 1.58
Akturk et al. [72]	Netherlands; DFU centre	Comparative cohort	Up to 1 year	*n* = 79 versus *n* = 271	ESRD: 7.7% versus 1.3%	1‐year healing: 53.2% → 76.4%	NR
Hartemann‐Heurtier et al. [73]	France; specialised MDT DFU unit	Observational cohort (prospective management)	7–29 months	*n* = 157 DFU; *n* = 118 analysed	RRT subgroup (size NR); RRT delays healing (*p* < 0.05)	50% (10 months); 70% (16 months); limb salvage 97.5%	NR
Ha Van et al. [74]	France; multidisciplinary DFU centre	Prospective single‐centre cohort	Median 19 months (12–24); ≥ 12 months follow‐up	*n* = 347 (*n* = 336 analysed)	Dialysis 13%; transplant 7%; high PAD (70%), infection (55%), osteomyelitis (47%)	Healing: 67% at 12 months; median healing time 6.6 months	NR
Altobelli et al. [75]	Italy; HD only	Prospective interventional	22 weeks (10 weeks + 3 months)	*n* = 25	100% HD	14.3% (10 weeks) → 57.1% (22 weeks)	NR
Meloni et al. [76]	Italy; tertiary DFU centre	Prospective cohort	Mean 15 ± 13 months	*n* = 599	Dialysis *n* = 99 (predominantly HD)	30.3% (dialysis) versus 52.6% (non‐dialysis)	NR
Messenger et al. [77]	Kuwait; DFU service	Retrospective cohort	Up to 6 months	*n* = 230 (335 DFUs)	CKD/ESKD subgroup (size NR)	ESKD ↓ healing (OR 0.3)	NR
Thai et al. [78]	Korea; surgical cohort	Retrospective cohort	NR	*n* = 65	ESRD subgroup (size NR)	Flap survival 91%	12.3%; ESRD OR 16.5
Utsunomiya et al. [79]	Japan; multicentre	Retrospective cohort	Mean 286 days	*n* = 431	Dialysis subgroup (size NR)	Healing 63.3%; dialysis ↓ healing	NR

Abbreviations: HD = haemodialysis; NR = not reported; PD = peritoneal dialysis; RRT = renal replacement therapy.

Patients with ESKD receiving HD demonstrate markedly poorer outcomes following lower limb revascularisation [81, 82], reflecting a distinct and more severe pattern of PAD [83, 84]. Dialysis populations demonstrate greater multivessel and below‐the‐ankle disease, with distal involvement strongly predicting revascularisation failure. Consequently, technical failure rates are substantially higher in dialysis populations (43.9% vs. 15.3%, *p* < 0.0001) [56, 84]. Overall outcomes of DFU in ESKD with HD remain poor, despite distal arterial revascularisation, with reduced graft patency at 1‐year (53% vs. 82% in non‐CKD) [85], higher perioperative mortality (9%) [56, 86], and frequent early amputations despite patent grafts [56, 87, 88]. Medium‐term survival is limited (∼33%–35% at 2 years) [84, 88, 89], regardless of whether revascularisation or primary amputation is performed [56, 84]. While selected patients—particularly those receiving vein grafts—may achieve reasonable short‐term limb salvage, this is often offset by high complication rates [81, 90] and prolonged hospitalisation [91]. Emerging strategies, including infra‐poplitaeal bypass and repeat endovascular interventions [71, 82, 92], may improve outcomes in selected cases, predominantly by reducing amputation rates [56, 82, 92, 93, 94]. However, the persistent gap between technical success and clinical outcomes highlights the profound systemic disease burden in ESKD and challenges the overall benefit and patient selection for revascularisation in this population [71, 81, 94].

### 3.7 Lower Extremity Amputation in ESKD and DFU

Although LEA is a key outcome in DFU, it remains a relatively infrequent event at the population level, with only 1.8% of DFU episodes resulting in major LEA within 6 months [54]. In contrast, individuals with ESKD—particularly those receiving dialysis—experience a markedly more aggressive disease course, with substantially higher rates of limb loss despite similar DFU incidence. Dialysis is consistently associated with a several‐fold increase in amputation risk, reflecting a shift in disease trajectory from ulceration to limb loss [42]. Compared with individuals with diabetes alone, those with ESKD have an approximately four‐fold higher risk of developing DFUs and a seven‐fold higher risk of major LEA [33]. Table 4 summarises LEA and mortality outcomes across mixed DFU, CKD, and dialysis cohorts, showing consistently higher amputation and death risks with worsening renal impairment, dialysis dependence, and advanced vascular disease.

**TABLE 4:** Lower‐extremity amputation (LEA) and mortality outcomes in DFU with ESKD/dialysis.

Study (author, year)	Country/setting	Study design	Follow‐up duration/study period	Population (N)	Dialysis/CKD subgroup	Comparator group	Lower‐extremity amputation (LEA)	Mortality
Franz et al. [24]	USA	Retrospective registry cohort	2000–2014	3,700,902 ESRD records	HD 92%; PD 8%; diabetes 54%	By diabetes, age, sex, region	LEA 4.09/100 PY (2014); major LEA fell 5.42 → 2.66/100 PY	1‐year mortality post‐LEA 52.2% → 43.6%
Harding et al. [25]	USA	Retrospective trend analysis	2000–2015	> 2 million ESRD + diabetes	HD & PD	Diabetes versus non‐diabetes ESRD	NLEA declined 7.5 → 4.2/100 PY, then plateaued	NR
Eggers et al. [95]	USA	Retrospective cohort	1991–1994	24,886 ESRD patients; 35,898 amputations	Dialysis + transplant ESRD population	General diabetic/non‐diabetic populations	LEA 11.8 → 13.8/100 PY in diabetic ESRD; 10× general diabetic population	50% at 1 year post‐LEA; 66% at 2 years
Tan et al. [26]	USA	Retrospective cohort	Mean 13.5 months	14,935	HD 92.5%; PD 7.4%; T2DM + new DFU	Podiatry versus no podiatry	Major amputation 28.3%; lower with podiatry care	Mortality 44.8%; lower composite death/amputation with podiatry
O'Hare et al. [96]	USA	Prospective registry‐linked cohort	2 years	9932	Dialysis 182 (1.8%)	By renal status	1‐year major LEA 29% in dialysis versus 10%–12% in other renal groups	1‐year mortality 35% in dialysis group
Speckman et al. [97]	USA	Retrospective cohort	12 months	3272	100% HD	Diabetes versus non‐diabetes	LEA incidence 4% at 12 months; diabetes HR 6.4	NR
Jaar et al. [98]	USA	Longitudinal cohort	3 years	800	100% HD after revascularisation	Bypass versus angioplasty	LEA 16.3/100 PY overall; higher with bypass 22.6 versus 5.7/100 PY	Higher mortality with bypass; RH 1.37 all‐cause
Sheahan et al. [99]	USA	Retrospective cohort	1990–2001	670	ESRD 11.5%	Minor amputation cohort	Major amputation conversion increased with ESRD (OR 1.72)	Survival 83.9% (1 year), 43.5% (5 years)
Owens et al. [81]	USA	Prospective registry cohort	1995–2004	456	CKD5/dialysis ∼15.7%	CKD stage	Major LEA higher in CKD5; limb salvage ∼50% in CKD5	5‐year survival 57%, 46%, 23%, 9.5% across CKD stages 1–5
Tarricone et al. [69] (IWJ)	USA	Retrospective cohort	1 year	344	eGFR ≥ 60 *n* = 219; 30–60 *n* = 63; < 30 *n* = 62	eGFR strata	No significant difference in amputation across eGFR groups	Mortality 1.9% versus 3.2% versus 8.1% by worsening eGFR
Tarricone et al. [100] (AVSG)	USA	Prospective cohort	NR	327	ESKD on HD/PD; PAD 88%; CAD 47%; DFU 36%	Controlled versus uncontrolled diabetes	HbA1c ≤ 6.5% associated with increased minor and major LEA	HbA1c ≤ 6.5% associated with lower mortality
Lavery et al. [42]	USA	Retrospective closed cohort	30 months pre/post dialysis	150	Dialysis cohort; modality NR	Pre‐ versus post‐dialysis; dialysis versus prior DFU cohort	First LEA 29.3 versus 37.3/1000 PY pre/post; dialysis versus prior DFU 58.0 versus 13.3/1000 PY	NR
Nandakumar et al. [88]	USA	Retrospective cohort	2010–2023	NR	ESKD subgroup	CKD + diabetes versus CKD without diabetes	CKD stage 5 + diabetes associated with ∼40‐fold higher major LEA	NR
Meloni et al. [101]	Italy	Observational cohort	1 year	1198	ESRD more common in ischaemic DFU	Neuropathic versus ischaemic DFU	Major LEA 0.5% versus 6.6%; ESRD independent predictor	Mortality 1.1% versus 11%
Meloni et al. [76]	Italy	Prospective cohort	Mean 15 ± 13 months	599	Dialysis 99 (predominantly HD)	Dialysis versus non‐dialysis	Major LEA 14.4% versus 10.8%; earlier in dialysis	21.1% versus 11%
Meloni et al. [102]	Italy	Retrospective observational	2019–2022	350	ESRD/dialysis predictor subgroup	With versus without in‐hospital complications	Major LEA 13.3% versus 3.1%	In‐hospital mortality 16.7% versus 0.6%
Meloni et al. [103]	Italy	Prospective cohort	1 year	239	ESRD more common in NO‐CLI	NO‐CLI versus ST‐CLI	Major LEA 30% versus 4.5%	Mortality 50% versus 8.9%
Meloni et al. [82]	Italy	Prospective cohort	1 year	136	Dialysis subgroup (R‐IF, HR‐IF)	IF versus HF versus dialysis versus HF + dialysis	Survival with major amputation: 6.1% IF, 7.7% H‐IF, 8.3% R‐IF, 10% HR‐IF	Mortality: 1.5%, 30.8%, 12.5%, 55%
Utsunomiya et al. [79]	Japan	Retrospective multicentre cohort	Mean 286 days	431	Dialysis subgroup present	None	Amputation 13.7%; dialysis independent predictor	Survival 87.7%; dialysis independent predictor of mortality
Honda et al. [71]	Japan	Observational cohort	Median 390–449 days	267	HD 120 versus non‐HD 147	HD versus non‐HD	Limb salvage 72.8% versus 86.4%, *p* = 0.002	NR
Orimoto et al. [104]	Japan	Retrospective cohort	1980–2011	234 (319 limbs)	100% HD	Dialysis‐only cohort	Major amputation in 37% of limbs	5‐year survival 23.4%; 7‐year survival 12.8%
Miyajima et al. [105]	Japan	Observational cohort	Mean ∼604 days	210	HD subgroup 30	Major amputation versus no major amputation	Major amputation 21.4%; haemodialysis HR 2.14	3‐year survival 24.1% major LEA versus 93.0% minor/non‐LEA
Holman et al. [27]	UK	Population‐based prospective cohort	2017–2022	71,000 new DFU	HD‐predominant RRT subgroup *n* = 2385 (3.4%); diabetes 100%; ischaemia recorded	None	NR	Mortality 4.2% (12 weeks), 8.2% (26 weeks), 14.4% (52 weeks); RRT independently associated with higher 26‐week mortality (RR 2.34, 95% CI 2.09–2.61)
Margolis et al. [35]	UK	Retrospective population cohort	Median 2.4 years	90,617	CKD spectrum; dialysis subset	CKD stage by eGFR	LEA HR 2.08 (eGFR 30–59); 7.71 (eGFR < 30)	NR
Game et al. [38]	UK	Self‐controlled case‐series	1976–2006	466	RRT cohort; modality NR	Pre‐ versus post‐dialysis initiation	Post‐RRT major LEA IR 31.98 (year 1), 34.0 (years 2–5)	NR
Ndip et al. [33]	UK	Cross‐sectional	Single assessment	326	CKD4–5 with/without dialysis	None	Prior amputation more common in dialysis: 15% versus 6.4%	NR
Paisey et al. [106]	UK	Retrospective cohort	2009–2011	250	CKD4–5 34	CKD4–5 versus CKD1–3	Major LEA 14.7% versus 4.6%	Mortality 29.4% versus 6.9%
Ndip et al. [107]	UK	Prospective cohort	2 years	192	100% dialysis + diabetes	DFU versus no DFU; prior LEA versus none	Baseline prior amputation in 35 patients	Mortality 53.1% overall; 59.3% with DFU; 74.3% with prior amputation
Shim et al. [108]	South Korea	Nationwide retrospective cohort	2002–2020	40,809	ESKD more prevalent in major LEA group	Major versus minor LEA	Major LEA group had higher ESKD prevalence (22.8% vs. 15.3%)	Mortality 73.9% major versus 52.2% minor LEA
Namgoong et al. [109]	South Korea	Retrospective cohort	2010–2013	860	Dialysis predictor subgroup	Major amputation versus none	Major LEA 3.3%; dialysis OR 8.68	NR
Seo et al. [56]	South Korea	Retrospective cohort	1998–2021	808	Dialysis prevalent; PD and HD subgroups	Limb salvage versus early major LEA	Major LEA 12.9%; CRF HR 2.97; PD HR 2.56	NR
Oh et al. [70]	South Korea	Retrospective surgical cohort	Mean 53.2 months	113	Renal failure/transplant subgroup present	None	Limb loss 17/113 (15.1%); limb salvage 84.9%	5‐year survival 86.8%
Thai et al. [78]	South Korea	Retrospective surgical cohort	NR	65	ESRD subgroup present	None	Amputation 2/65 (3.1%)	NR
Rubio et al. [93]	Spain	Retrospective cohort	Up to 12.2 years	338	CKD subgroup present	None	Major LEA not explicitly reported	Overall mortality 59.5%; CKD HR 1.86
Dòria et al. [44]	Spain	Retrospective cohort	∼5 years	220	100% HD	Diabetes versus non‐diabetes; DFS versus no DFS	LEA/new FU higher in diabetes; HR ∼2.42	55.9% at 5 years; higher in diabetes
Felipe et al. [45]	Spain	Cross‐sectional	2017–2018	104	CKD stage V 88%; dialysis 73%	None	Amputation prevalence 8%	NR
Dòria et al. [41]	Spain	Cross‐sectional	Single assessment	92	Mixed HD/PD	None	Prior amputation 16.3%	NR
Hamilton et al. [110] (FDS I & II)	Australia	Prospective longitudinal cohort	Up to 5 years	1296; 1509	Community T2DM cohort; ESKD in predictor model	FDS2 versus FDS1	Minor LEA 23.7 versus 14.1/10,000 PY; major LEA 15.1 versus 9.8/10,000 PY; ESKD HR 28.5 unadjusted, 35.6 adjusted	Mortality as competing outcome; no specific rate
Lan et al. [111]	Australia	Prospective observational cohort	Median ∼410 days	497	CKD defined as eGFR < 60	SINBAD/CKD strata	Not reported	MACE/all‐cause mortality 5.5% → 53.7% across severity groups
Kaminski et al. [37]	Australia	Multicentre prospective cohort	12 months	450	HD 94%; PD 6%; diabetes 50%	With versus without DFU	2.7% (12 amputations)	11.6% (52 deaths)
Stuart et al. [112]	Australia	Retrospective audit	2012–2017	166	Dialysis common in Aboriginal subgroup	Aboriginal versus non‐Aboriginal; dialysis versus non‐dialysis	Dialysis associated with higher repeat amputation	Higher mortality in dialysis patients post‐amputation
Wolf et al. [113]	Germany	Retrospective cohort	1989–2007	4906	CKD spectrum	DFS versus no DFS; CKD stages	LEA not directly quantified; worsening CKD associated with more severe DFS	NR
Ogurtsova et al. [68]	Germany/Czech Republic	Prospective cohort	Up to 15 years	321	ESKD on RRT subgroup	None	Minor LEA associated with higher recurrence in CZ (HR 2.11)	25% (GER) and 15% (CZ) died without recurrence
Morbach et al. [52]	Germany	Prospective cohort	> 10 years	247	Dialysis/CKD subgroup present	None	First major LEA 15.4%; 22.3% cumulative at 10 years; dialysis HR 3.51	Mortality 15.4% (1 year), 33.1% (3 years), 45.8% (5 years), 70.4% (10 years); dialysis HR 6.43
Bonnet et al. [30]	France	Nationwide retrospective cohort	1 year	133,791	ESKD identified as risk factor	With versus without major amputation	Major amputation 3.5%; ESKD HR 2.12	NR
Hartemann‐Heurtier et al. [73]	France	Observational cohort	7–29 months	157; 118 analysed	RRT subgroup present	None	Limb salvage 97.5%	NR
Otte et al. [34]	Netherlands	Retrospective cohort	2006–2012	669	Dialysis 259	CKD3 versus CKD4–5 versus dialysis	Major amputation HR 9.5 (CKD4–5) and 15.0 (dialysis) versus CKD3	NR
Akturk et al. [72]	Netherlands	Comparative cohort	2003–2004 versus 2014–2018	79 versus 271	ESRD 1.3% versus 7.7%	Historical versus contemporary cohorts	Minor LEA 20.3% versus 8.1%; major LEA 5.1% versus 3.0%	12‐month mortality 15.2% versus 13.7%
He et al. [114]	China	Prospective cohort	Mean 37 months	366	CKD spectrum	eGFR ≥ 90 reference	LEA not explicit; healing failure HR 2.08 and 2.84 in moderate/severe CKD	Mortality HR 3.54 and 4.45 in moderate/severe CKD
Zhang et al. [115]	China	Prospective cohort	6 months	321	eGFR categories; severe CKD subgroup	eGFR strata	Total amputation 65.1%; severe CKD associated with minor LEA OR 4.05 and total amputation OR 4.50	Minimal mortality; no formal analysis
Combe et al. [28] (DOPPS)	Multinational	Prospective observational cohort	Mean 1.47 years	29,838	100% HD; diabetes 37.3%	Diabetes versus non‐diabetes; country comparisons	2.0 amputations/100 PY overall; 4.6 versus 0.5/100 PY in diabetes versus non‐diabetes	Post‐LEA mortality RR 1.54
Prompers et al. [116]	Europe	Prospective cohort	1 year	1088	ESKD 5.8%	PAD versus non‐PAD	Major LEA 5% overall; 8% PAD versus 2% no PAD	Mortality 6% overall; 9% PAD versus 3% no PAD
Randon et al. [117]	Europe	Retrospective cohort	1993–2007	76	Dialysis 3; CKD 32	None	Early major LEA 6%; additional 21% later; renal insufficiency OR 16.3	In‐hospital mortality 3.8%; survival + limb salvage 51% at 5 years
Al‐Thani et al. [40]	Qatar	Retrospective cohort	5 years	252	100% HD; PAD 41%	Foot ulcer versus no foot ulcer	PAD‐associated major LEA 14% versus 2%	56.7% over 5 years; DFU aOR 3.6
Tay et al. [118]	Singapore	Retrospective cohort	2 years	207	ESRF predictor subgroup	Risk factor analysis	2‐year major LEA 30%; ESRF associated with worse limb outcomes	Mortality 7.7% (1 month), 16.4% (6 months), 21.7% (12 months)
Spinelli et al. [43]	Argentina	Cross‐sectional	2022–2024	54	HD 73%; PD 27%	HD versus PD	Prior LEA 31.4%; major LEA 5.6%; no modality difference	NR
Adeleye et al. [119]	Nigeria	Prospective observational	NR	112	Renal impairment/dialysis subgroup	None	24.7% amputated	18.4%; associated with renal impairment/dialysis
Akha et al. [120]	Iran	Retrospective cohort	1996–2005	244	Renal dysfunction subgroup	Amputees versus non‐amputees	27.0% amputated; higher creatinine in amputees	NR
Alashek et al. [121]	Libya	Cross‐sectional	2009	749	99.5% HD	None	Amputation prevalence 9.1%	NR
Venermo et al. [94]	Finland	Retrospective cohort	1 year	597 (732 revascularisations)	Dialysis subset 9.2%	CKD stage; procedure type	CKD class independently predicted major LEA/limb salvage	NR

Abbreviations: HD = haemodialysis; NR = not reported; PD = peritoneal dialysis; RRT = renal replacement therapy.

In a retrospective cohort study by Lavery et al. [42], approximately one quarter of patients developed a DFU over 30 months. Despite similar ulcer incidences, dialysis‐treated individuals experienced substantially higher and more recurrent amputation events [42]. First‐amputation incidence was three‐fold higher in dialysis patients than in those with prior DFU (32.0 vs. 10.7 per 1000 person‐years), while cumulative LEA rates were more than four‐fold higher (58.0 vs. 13.1 per 1000 person‐years).

In a prospective cohort study by Morbach et al. [52], long‐term outcomes (up to 15 years) in patients with DFU demonstrated a strong association between ESKD and LEA. Dialysis emerged as an independent predictor of LEA [52], even after adjustment for key confounders, indicating that ESKD directly contributes to adverse limb outcomes rather than acting solely as a marker of disease severity. Notably, amputation events were overwhelmingly driven by coexisting PAD, highlighting the central role of ischaemia in this population.

Findings from the Fremantle Diabetes Study further reinforce the impact of ESKD on LEA risk [110]. Although overall rates of both minor and major amputations declined over time, these differences were not statistically significant. In contrast, ESKD emerged as a powerful independent predictor of major limb loss [110], with a markedly elevated risk in Cox models (HR ∼28.5), which remained robust after adjustment (HR ∼35.6, *p* < 0.001) [110]. These effect sizes substantially exceed those of traditional risk factors, highlighting the disproportionate burden of limb loss in this subgroup. Collectively, these data indicate that, despite improvements in population‐level outcomes, ESKD defines a cohort with persistently high amputation risk, reflecting severe systemic vascular disease and impaired capacity for limb salvage.

Across observational cohorts, ESKD consistently emerges as one of the strongest independent predictors of major LEA, even after adjustment for age, diabetes duration, and cardiovascular comorbidity [35, 122]. A clear gradient of DFU risk exists across stages of CKD, with risk rising progressively from moderate CKD and accelerating sharply once eGFR falls below 30 mL/min/1.73 m^2^ and after dialysis initiation [35]. This dose–response relationship, demonstrated in longitudinal and adjusted analyses [34], supports renal impairment as an independent and biologically relevant driver of foot pathology rather than a surrogate for diabetes severity, with advancing CKD amplifying vascular, neuropathic, and inflammatory mechanisms that increase ulcer severity, recurrence, and amputation risk [34].

National dialysis datasets [122, 123] demonstrate that DFU in ESKD with dialysis cohorts is frequently followed by major LEA within the first and second year of initiating dialysis [26]. Ulcer severity features—especially ischaemia, infection [124], and depth—are strong predictors of limb loss [125, 126]. Importantly, even when technically successful revascularisation is achieved, limb salvage rates remain poor in ESKD, highlighting the limitations of conventional vascular interventions in the presence of CKD‐related microvascular dysfunction, inflammation, and frailty [127].

### 3.8 Mortality in ESKD With DFU

Mortality outcomes in patients with DFU and ESKD receiving dialysis are summarised in Table 4 and demonstrate a markedly worse survival trajectory than the general DFU population. While 5‐year survival among people with DFU is around 70%, this declines to approximately 43% following major transtibial amputation [128], and to under 10% in patients with ESKD [129], compared with nearly 70% in those without renal failure [128]. Taken together, most of the observational evidence derived from national‐registry data and large cohort studies indicates that DFU in the context of HD identifies a population with extreme systemic risk [28, 76], in where LEA is the manifestation of a complication which precedes a terminal event. While precise mortality estimates vary by study design, one‐year mortality commonly exceeds 10%–20%, and three‐to 5‐year mortality approaches or exceeds 50%–70%, particularly following major LEA.

Data from the National Diabetes Foot Care Audit (NDFA) for England and Wales provide robust population‐level evidence of mortality following DFU [27]. Among more than 71,000 individuals with a newly registered DFU between 2017 and 2022, mortality reached 4.2% at 12 weeks, 8.2% at 26 weeks, and 14.4% at 52 weeks [27]. Renal replacement therapy was one of the strongest independent predictors of death, conferring more than a 2‐fold increase in 26‐week mortality risk (rate ratio 2.34) [27]. However, as these findings are derived from adjusted odd ratios, absolute probabilities of combined survival and healing cannot be directly inferred.

Despite variability in ulcer severity reporting across centres, the magnitude and consistency of excess mortality in ESKD populations are observed across multiple datasets [130]. CKD3‐5 (eGFR < 30) independently predicts death, with risk rising sharply at advanced stages and after dialysis initiation [20, 35, 122]. Patients with ESKD demonstrated markedly poorer limb salvage following revascularisation, with substantially higher rates of major LEA compared with lower CKD stages, even in the presence of patent grafts [81]. Limb outcomes deteriorated progressively with worsening renal function, highlighting the limited durability of revascularisation in advanced CKD [34, 81]. Mortality was similarly poor, with 5‐year survival falling to < 10% in CKD‐5 [28, 76], confirming ESKD as a strong independent predictor of death following LEA [76].

Analyses from the US Renal Data System demonstrate very high mortality among dialysis patients presenting with a new DFU [131], with 5‐year survival estimated at approximately 40% [132, 133]. The majority experience death or major amputation within 13–14 months, and three‐year mortality approaches 70% [26]. Despite reliance on administrative claims data, which may be subject to delayed reporting and misclassification, the consistency and scale of this signal across datasets strongly support DFU in ESKD as a marker of severe systemic disease rather than an isolated limb complication [123]. In addition, cause‐of‐death data and underlying kidney disease classifications may be inaccurate or fail to reflect changes in clinical status over time [134]. These limitations likely attenuate, rather than exaggerate, observed mortality estimates [37]. Mortality clustered among individuals with established neuropathy, PAD and prior ulceration [37], reinforcing the concept that DFU identifies a subgroup with markedly elevated systemic risk rather than acting as an isolated determinant of death [135, 136, 137].

In the DOPPS study [28], diabetes was associated with a nine‐fold higher incidence of LEA. Amputation independently doubled the risk of death (HR 1.54; 95% CI 1.41–1.68), reducing mean survival from 3.8 to 2.0 years [28]. Although DFU severity and timing relative to amputation were not characterised, the multinational scope and prospective design of this study underscore the lethality of limb loss in ESKD. Patient‐level studies highlight a striking discordance between perceived and actual risk. In a cohort of 461 people with diabetes, including 48 patients with ESKD receiving HD, individuals with diabetic foot disease were more than twice as likely to rank major LEA as their greatest fear and less likely to fear death than those without foot complications [138]. Yet 5‐year mortality was more than twice as high in the ESKD group (52.4% vs. 23.5%) despite similar stump healing rates. Functional outcomes diverged sharply: fewer than half of ESKD patients remained ambulatory, and loss of ambulation independently predicted mortality [138].

Evidence from predominantly observational cohort [104] and registry studies [25, 139] consistently indicates that dialysis‐dependent ESKD is associated with poor long‐term outcomes, including reduced survival and a high burden of limb loss. Large national registry data [25, 27, 28, 91]provide relatively robust evidence, demonstrating persistently elevated rates of non‐traumatic LEA in individuals with diabetes and ESKD [140] compared with diabetes alone [27, 141, 142], although trends have plateaued in recent years despite earlier declines [25, 143]. While such datasets offer strong external validity due to scale, their reliance on administrative coding and limited clinical granularity introduces potential misclassification and residual confounding. Across studies using adjusted analyses, dialysis dependence and ESKD consistently emerged as independent risk factors for amputation after accounting for key confounders [37, 88]. Smaller cohort studies ^110^further support these findings, identifying dialysis and chronic renal failure as significant predictors of early major amputation [39, 107, 144], although generalisability is limited by single‐centre designs. Despite heterogeneity in study populations and methodologies [145], the direction of effect is consistent, suggesting a meaningful and independent contribution of dialysis‐dependent ESKD to adverse limb outcomes. Collectively, these findings indicate that dialysis dependence is associated with greater amputation risk, reduced mobility, and poorer overall outcomes, although conclusions are constrained by the observational nature of the evidence.

### 3.9 Prevention and Foot Surveillance in Dialysis Populations

A study from the Fresenius Dialysis Centres Network in North America demonstrated that implementation of a structured foot‐care programme delivered by dedicated dialysis nursing staff was associated with a significant reduction in major lower‐extremity amputations, decreasing from 1.30 to 1.07 per 100 person‐years [19]. These findings highlight the potential benefit of proactive foot surveillance, glycaemic management and multidisciplinary care within dialysis services [69, 100, 124]. Consistent with these findings, several interventional studies suggest that targeted foot‐care strategies in dialysis populations can improve clinical outcomes, particularly when tailored to high‐risk groups and specific ethnic subpopulations [146, 147, 148].

### 3.10 Gaps in Clinical Research Evidence

Despite the high burden of DFU in people with ESKD, the clinical trial evidence base remains fragmented and poorly adapted to this population. A central limitation is the systematic exclusion or under‐representation of dialysis patients in DFU randomised controlled trials, particularly those evaluating pharmacotherapies and advanced wound therapies. Important knowledge gaps also exist in vascular and infection‐related management. Patients receiving dialysis frequently present with advanced PAD and complex tissue loss, yet RCT evidence guiding optimal revascularisation strategies or dialysis‐adjusted antibiotic regimens remains limited. Similarly, offloading strategies have rarely been evaluated within the physiological and logistical context of dialysis care, where intradialytic haemodynamic shifts, prolonged sitting during treatment, altered biomechanics, and increased falls risk may influence wound healing and adherence.

## 4 Discussion

This review synthesised evidence from 64 observational studies and registry datasets, demonstrating a high burden of DFU in adults with ESKD receiving dialysis, with elevated risks of major amputation and mortality. Dialysis emerges as a key determinant of adverse limb and survival outcomes, although findings are limited by heterogeneity and observational study designs. Evidence regarding DFU incidence at dialysis initiation remains inconsistent, likely reflecting variation in definitions, study populations, and analytical approaches [38, 42].

All included studies were observational, with inherent risks of bias, confounding, and variability in outcome reporting. Representation of dialysis populations varied widely, with haemodialysis‐dominant cohorts in some studies and under‐representation in large registries, limiting comparability. Follow‐up durations were typically short (≤ 1–2 years), limiting assessment of long‐term outcomes, particularly ulcer recurrence, which was infrequently reported.

Most data originated from UK and US settings, primarily from registry‐based or specialist care cohorts [53]. However, meaningful cross‐country comparisons are limited. Differences in healthcare systems —universal audit‐driven care within the UK National Health Service versus insurance‐based models in the US—affect access, case ascertainment, and data capture. Registry methodologies also differ, with administrative coding in US datasets (e.g., United States Renal Data System) contrasting with clinically curated UK audit data (e.g., National Diabetes Foot Care Audit). These structural and methodological differences introduce significant confounding factors and preclude robust direct comparisons. Standardised international datasets and prospective studies are required to enable valid comparative analyses.

Centre‐based haemodialysis populations predominated, reflecting current service delivery models in the included studies, particularly in the UK and the United States, where home haemodialysis remains comparatively uncommon. Patients receiving home haemodialysis may represent a less multimorbid subgroup with a potentially different DFU risk profile, introducing possible selection bias. However, these populations were not specifically identified or stratified in the available literature, limiting conclusions regarding comparative risk. This remains an important evidence gap warranting targeted investigation.

Several additional limitations should be acknowledged. Broad inclusion criteria required adjudication in some cases, and it is unlikely that all relevant studies were captured. Subgroup analyses within dialysis populations were not explored, despite recognised variability in risk profiles. Future research should focus on stratified analyses to better inform targeted prevention and management strategies.

DFU in dialysis‐dependent ESKD is associated with a convergence of adverse outcomes, including impaired healing, high amputation rates, and elevated mortality [57]. Across studies, these complications are rarely isolated; rather, they occur concurrently and interactively, reflecting the complex interplay between neuropathy, PAD, metabolic dysregulation, and systemic illness. These findings support the concept that DFU in dialysis‐dependent ESKD represents an accelerated and high‐risk phenotype, characterised by poor healing, early recurrence, and a strong ischaemic component driven by coexisting PAD [149].

Evidence on whether dialysis initiation increases DFU risk is limited and heterogeneous. Observational cohorts suggest a temporal association with haemodialysis initiation [38], particularly within the first 2 years; however, data also indicate that DFU risk is established prior to renal replacement therapy and increases with declining kidney function [30, 31]. Longitudinal studies report no consistent rise in ulcer or amputation incidence following dialysis initiation [35], with some showing stable or lower post‐dialysis ulcer burden [42], although findings are constrained by observational design and potential confounding factors. These patterns suggest that dialysis may represent a period of heightened vulnerability [150] rather than a discrete inflection point in risk, but the underlying mechanisms [151, 152, 153] and their relative contributions remain uncertain [69, 154, 155].

The evidence synthesised in this review highlights important gaps in understanding risk stratification and disease progression in dialysis‐dependent ESKD. Data are limited and heterogeneous regarding the temporal relationship between dialysis initiation and DFU risk, with inadequate stratification by dialysis modality and inconsistent reporting of key outcomes such as healing durability and recurrence [156]. Furthermore, few studies examine early or pre‐ulcer risk trajectories, limiting insight into optimal timing for intervention. These gaps underscore the need for prospective, dialysis‐specific studies to better define risk progression and inform targeted preventive strategies. Future research must prioritise ESKD populations—historically underrepresented in clinical studies—to better define mechanistic pathways, refine risk stratification, and inform targeted multidisciplinary prevention strategies aimed at reducing both limb loss and premature mortality.

## Author Contributions

**J. Z. M. Lim:** conceptualization, data acquisition, analysis and interpretation, drafting of the manuscript, critical revision and editing. **D. Selvarajah:** data acquisition, analysis and interpretation, critical revision and editing. **S. Mitra:** critical revision and editing. **N. S. L. Ng:** data analysis, interpretation, critical revision and editing. **G. Rayman:** critical revision and editing. **L. Vileikyte:** critical revision and editing. **F. L. Game:** data analysis, interpretation, critical revision and editing. **A. J. M. Boulton:** conceptualization, data analysis, interpretation, critical revision and editing.

## Funding

The authors have nothing to report.

## Conflicts of Interest

The authors declare no conflicts of interest.

## Supporting information


Supporting Information S1

Supporting Information S2

Supporting Information S3

**Table S1:** STROBE scoring framework.

## Data Availability

The data that support the findings of this study are available from the corresponding author upon reasonable request.

## References

[1] B. Z. Chin , P. Lee , C. H. Sia , and C. C. Hong , “Diabetic Foot Ulcer Is Associated With Cardiovascular‐Related Mortality and Morbidity—A Systematic Review and Meta‐Analysis of 8062 Patients,” Endocrine 84, no. 3 (June 2024): 852–863, 10.1007/s12020-024-03696-5. 38280983

[2] I. H. de Boer , K. Khunti , T. Sadusky , et al., “Diabetes Management in Chronic Kidney Disease: A Consensus Report by the American Diabetes Association (ADA) and Kidney Disease: Improving Global Outcomes (KDIGO),” Diabetes Care 45, no. 12 (December 2022): 3075–3090, 10.2337/dci22-0027. 36189689 PMC9870667

[3] J. Jankowski , J. Floege , D. Fliser , M. Böhm , and N. Marx , “Cardiovascular Disease in Chronic Kidney Disease: Pathophysiological Insights and Therapeutic Options,” Circulation 143, no. 11 (March 2021): 1157–1172, 10.1161/circulationaha.120.050686. 33720773 PMC7969169

[4] D. N. Koye , D. J. Magliano , R. G. Nelson , and M. E. Pavkov , “The Global Epidemiology of Diabetes and Kidney Disease,” Advances in Chronic Kidney Disease 25, no. 2 (March 2018): 121–132, 10.1053/j.ackd.2017.10.011. 29580576 PMC11000253

[5] D. G. Armstrong , A. J. M. Boulton , and S. A. Bus , “Diabetic Foot Ulcers and Their Recurrence,” New England Journal of Medicine 376, no. 24 (June 2017): 2367–2375, 10.1056/NEJMra1615439. 28614678

[6] GBD 2021 Diabetes Collaborators , “Global, Regional, and National Burden of Diabetes From 1990 to 2021, With Projections of Prevalence to 2050: A Systematic Analysis for the Global Burden of Disease Study 2021,” Lancet 402, no. 10397 (July 2023): 203–234, 10.1016/s0140-6736(23)01301-6. 37356446 PMC10364581

[7] A. J. M. Boulton and R. W. Whitehouse , “The Diabetic Foot,” in Endotext, ed. K. R. Feingold , B. Anawalt , M. R. Blackman , et al., (MDText.com, Inc., 2023), https://www.ncbi.nlm.nih.gov/books/NBK409609/.

[8] D. G. Armstrong , T. W. Tan , A. J. M. Boulton , and S. A. Bus , “Diabetic Foot Ulcers: A Review,” JAMA 330, no. 1 (July 2023): 62–75, 10.1001/jama.2023.10578. 37395769 PMC10723802

[9] K. Ismail , K. Winkley , D. Stahl , T. Chalder , and M. Edmonds , “A Cohort Study of People With Diabetes and Their First Foot Ulcer: The Role of Depression on Mortality,” Diabetes Care 30, no. 6 (2007): 1473–1479, 10.2337/dc06-2313. 17363754

[10] L. M. Brooks , B. M. Brooks , A. S. Arp , et al., “Diabetes‐Related Extremity Amputation Depression and Distress (DREADD): A Multimethod Study,” Seminars in Vascular Surgery 38, no. 1 (March 2025): 94–100, 10.1053/j.semvascsurg.2025.01.002. 40086927

[11] J. Chilcot , A. Guirguis , K. Friedli , et al., “Depression Symptoms in Haemodialysis Patients Predict All‐Cause Mortality But Not Kidney Transplantation: A Cause‐Specific Outcome Analysis,” Annals of Behavioral Medicine 52, no. 1 (January 2018): 1–8, 10.1007/s12160-017-9918-9. 28762106 PMC6367894

[12] V. Fejfarová , A. Jirkovská , E. Dragomirecká , et al., “Does the Diabetic Foot Have a Significant Impact on Selected Psychological or Social Characteristics of Patients With Diabetes Mellitus?,” Journal of Diabetes Research 2014 (2014): 371938, 10.1155/2014/371938. 24791012 PMC3984852

[13] M. M. Iversen , G. S. Tell , B. Espehaug , et al., “Is Depression a Risk Factor for Diabetic Foot Ulcers? 11‐Years Follow‐Up of the Nord‐Trøndelag Health Study (HUNT),” Journal of Diabetic Complications 29, no. 1 (January–February 2015): 20–25, 10.1016/j.jdiacomp.2014.09.006. 25283486

[14] S. Kim , J. Jeon , Y.‐J. Lee , et al., “Depression is a Main Determinant of Health‐Related Quality of Life in Patients With Diabetic Kidney Disease,” Scientific Reports 12, no. 1 (2022/07/16 2022): 12159, 10.1038/s41598-022-15906-z. PMC9288542 35842489

[15] C. M. Morsch , L. F. Gonçalves , and E. Barros , “Health‐Related Quality of Life Among Haemodialysis Patients—Relationship With Clinical Indicators, Morbidity and Mortality,” Journal of Clinical Nursing 15, no. 4 (April 2006): 498–504, 10.1111/j.1365-2702.2006.01349.x. 16553764

[16] Y. Benhamou , L. Begarin , N. David , et al., “Detection of Microcirculatory Impairment by Transcutaneous Oxymetry Monitoring During Hemodialysis: An Observational Study,” BMC Nephrology 15, no. 1 (January 2014): 4, 10.1186/1471-2369-15-4. 24400914 PMC3906095

[17] J. E. Flythe , H. Xue , K. E. Lynch , G. C. Curhan , and S. M. Brunelli , “Association of Mortality Risk With Various Definitions of Intradialytic Hypotension,” Journal of the American Society of Nephrology 26, no. 3 (March 2015): 724–734, 10.1681/asn.2014020222. 25270068 PMC4341481

[18] J. J. Sands , L. A. Usvyat , T. Sullivan , et al., “Intradialytic Hypotension: Frequency, Sources of Variation and Correlation With Clinical Outcome,” Hemodialysis International 18, no. 2 (April 2014): 415–422, 10.1111/hdi.12138. 24467830

[19] P. A. Marn , V. Peršič , L. Usvyat , et al., “Implementation of Routine Foot Check in Patients With Diabetes on Hemodialysis: Associations With Outcomes,” BMJ Open Diabetes Research & Care 4, no. 1 (2016): e000158, 10.1136/bmjdrc-2015-000158. PMC4780043 26958348

[20] N. S. R. Lan , G. Dwivedi , P. G. Fegan , F. Game , and E. J. Hamilton , “Unravelling the Cardio‐Renal‐Metabolic‐Foot Connection in People With Diabetes‐Related Foot Ulceration: A Narrative Review,” Cardiovascular Diabetology 23, no. 1 (December 2024): 437, 10.1186/s12933-024-02527-1. 39696281 PMC11657306

[21] N. Schembri and C. Formosa , “Dialysis Treatment Is an Independent Risk Factor for Foot Morbidity,” International Journal of Lower Extremity Wounds 23, no. 4 (December 2024): 533–540, 10.1177/15347346221074111. 35037518

[22] K. Sandepudi , K. V. Shah , B. A. Melnick , et al., “Pathophysiology of Wound Development and Chronicity in Renal Disease: A Narrative Review,” International Wound Journal 22, no. 7 (July 2025): e70713, 10.1111/iwj.70713. 40579936 PMC12205259

[23] L. Årestedt , C. Martinsson , C. Hjelm , F. Uhlin , and A. C. Eldh , “Patient Participation in Dialysis Care‐A Qualitative Study of Patients' and Health Professionals' Perspectives,” Health Expectations 22, no. 6 (December 2019): 1285–1293, 10.1111/hex.12966. 31560830 PMC6882253

[24] D. Franz , Y. Zheng , N. J. Leeper , V. Chandra , M. Montez‐Rath , and T. I. Chang , “Trends in Rates of Lower Extremity Amputation Among Patients With End‐Stage Renal Disease Who Receive Dialysis,” JAMA Internal Medicine 178, no. 8 (August 2018): 1025–1032, 10.1001/jamainternmed.2018.2436. 29987332 PMC6143114

[25] J. L. Harding , M. E. Pavkov , E. W. Gregg , and N. R. Burrows , “Trends of Nontraumatic Lower‐Extremity Amputation in End‐Stage Renal Disease and Diabetes: United States, 2000–2015,” Diabetes Care 42, no. 8 (August 2019): 1430–1435, 10.2337/dc19-0296. 31142496 PMC11000250

[26] T. W. Tan , B. Caldwell , Y. Zhang , O. Kshirsagar , D. J. Cotter , and T. W. Brewer , “Foot and Ankle Care by Podiatrists and Amputations in Patients With Diabetes and Kidney Failure,” JAMA Network Open 7, no. 3 (March 2024): e240801, 10.1001/jamanetworkopen.2024.0801. 38427353 PMC10907919

[27] N. Holman , A. C. Yelland , B. Young , J. Valabhji , W. Jeffcoate , and F. Game , “Mortality Rates in People Presenting With a New Diabetes‐Related Foot Ulcer: A Cohort study With Implications for Management,” Diabetologia 67, no. 12 (December 2024): 2691–2701, 10.1007/s00125-024-06262-w. 39331060 PMC11604764

[28] C. Combe , J. M. Albert , J. L. Bragg‐Gresham , et al., “The Burden of Amputation Among Hemodialysis Patients in the Dialysis Outcomes and Practice Patterns Study (DOPPS),” American Journal of Kidney Diseases 54, no. 4 (October 2009): 680–692, 10.1053/j.ajkd.2009.04.035. 19619923

[29] N. S. Skolnik and A. J. Style , “Importance of Early Screening and Diagnosis of Chronic Kidney Disease in Patients With Type 2 Diabetes,” Diabetes Therapy 12, no. 6 (June 2021): 1613–1630, 10.1007/s13300-021-01050-w. 33914300 PMC8179861

[30] J.‐B. Bonnet , C. Duflos , H. Huguet , A. Avignon , and A. Sultan , “Epidemiology of Major Amputation Following Diabetic Foot Ulcer: Insights From Recent Nationwide Data in the French National Health Registry (SNDS),” Diabetes & Metabolism 51, no. 2 (2025): 101606, 10.1016/j.diabet.2025.101606. 39814334

[31] J. B. Bonnet , I. Szwarc , A. Avignon , S. Jugant , and A. Sultan , “Renal Function Is Highly Associated With Podiatric Risk in Diabetic Patients,” Clinical Kidney Journal 16, no. 11 (November 2023): 2156–2163, 10.1093/ckj/sfad106. 37915919 PMC10616501

[32] Z. Zhang , T. Cui , M. Cui , and X. Kong , “High Prevalence of Chronic Kidney Disease Among Patients With Diabetic Foot: A Cross‐Sectional Study at a Tertiary Hospital in China,” Nephrology 25, no. 2 (February 2020): 150–155, 10.1111/nep.13596. 31025471

[33] A. Ndip , M. K. Rutter , L. Vileikyte , et al., “Dialysis Treatment Is an Independent Risk Factor for Foot Ulceration in Patients With Diabetes and Stage 4 or 5 Chronic Kidney Disease,” Diabetes Care 33, no. 8 (August 2010): 1811–1816, 10.2337/dc10-0255. 20484126 PMC2909067

[34] J. Otte , J. J. van Netten , and A. J. Woittiez , “The Association of Chronic Kidney Disease and Dialysis Treatment With Foot Ulceration and Major Amputation,” Journal of Vascular Surgery 62, no. 2 (August 2015): 406–411, 10.1016/j.jvs.2015.02.051. 25937604

[35] D. J. Margolis , O. Hofstad , and H. I. Feldman , “Association Between Renal Failure and Foot Ulcer or Lower‐Extremity Amputation in Patients With Diabetes,” Diabetes Care 31, no. 7 (July 2008): 1331–1336, 10.2337/dc07-2244. 18390800 PMC2453658

[36] N. C. Schaper , J. J. van Netten , J. Apelqvist , et al., “Practical Guidelines on the Prevention and Management of Diabetes‐Related Foot Disease (IWGDF 2023 Update),” Diabetes/Metabolism Research and Reviews 40, no. 3 (March 2024): e3657, 10.1002/dmrr.3657. 37243927

[37] M. R. Kaminski , K. A. Lambert , A. Raspovic , et al., “Risk Factors for Foot Ulceration in Adults With End‐Stage Renal Disease on Dialysis: A Prospective Observational Cohort Study,” BMC Nephrology 20, no. 1 (November 2019): 423, 10.1186/s12882-019-1594-5. 31752749 PMC6868750

[38] F. L. Game , S. Y. Chipchase , R. Hubbard , R. P. Burden , and W. J. Jeffcoate , “Temporal Association Between the Incidence of Foot Ulceration and the Start of Dialysis in Diabetes Mellitus,” Nephrology Dialysis Transplantation 21, no. 11 (2006): 3207–3210, 10.1093/ndt/gfl427. 16877485

[39] L. A. Lavery , D. C. Lavery , N. A. Hunt , J. La Fontaine , A. Ndip , and A. J. Boulton , “Amputations and Foot‐Related Hospitalisations Disproportionately Affect Dialysis Patients,” International Wound Journal 12, no. 5 (October 2015): 523–526, 10.1111/iwj.12146. 24103293 PMC7950888

[40] H. Al‐Thani , A. El‐Menyar , V. Koshy , et al., “Implications of Foot Ulceration in Hemodialysis Patients: A 5‐Year Observational Study,” Journal of Diabetes Research 2014 (2014): 945075–945076, 10.1155/2014/945075. 24724108 PMC3958654

[41] M. Dòria , V. Rosado , L. R. Pacheco , et al., “Prevalence of Diabetic Foot Disease in Patients With Diabetes Mellitus Under Renal Replacement Therapy in Lleida, Spain,” Biomed Research International 2016 (2016): 7217586–7217588, 10.1155/2016/7217586. 27190996 PMC4848423

[42] L. A. Lavery , D. C. Lavery , N. A. Hunt , J. Fontaine , and R. D. Lavery , “Does the Start of Dialysis Initiate a Period of Increased Risk of Ulceration or Amputation?,” Journal of the American Podiatric Medical Association 108, no. 1 (January 2018): 1–5, 10.7547/16-056. 29547031

[43] M. B. Spinelli , V. M. Cafruni , N. Lucero Viviani , et al., “Prevalence of Diabetic Foot in Patients With Diabetes Mellitus Undergoing Dialysis Treatment in a Tertiary‐Level Hospital in Argentina,” Revista Española de Cirugía Ortopédica y Traumatología 70, no. 1 (2025): 8–12, 10.1016/j.recot.2025.04.018. 42024520

[44] M. Dòria , A. Betriu , M. Belart , et al., “High Incidence of Adverse Outcomes in Haemodialysis Patients With Diabetes With or Without Diabetic Foot Syndrome: A 5‐Year Observational Study in Lleida, Spain,” Journal of Clinical Medicine 10, no. 7 (2021): 1368, 10.3390/jcm10071368. 33810545 PMC8037880

[45] R. R. Felipe , “Risk Factors for Foot Problems Among Patients With Type 2 Diabetes and Chronic Kidney Disease,” supplement, Journal of the Endocrine Society 3, no. S1 (2019), 10.1210/js.2019-SAT-135.

[46] F. Crawford , C. McCowan , B. D. Dimitrov , et al., “The Risk of Foot Ulceration in People With Diabetes Screened in Community Settings: Findings From a Cohort Study,” QJM 104, no. 5 (May 2011): 403–410, 10.1093/qjmed/hcq227. 21186178

[47] C. A. Abbott , A. L. Carrington , H. Ashe , et al., “The North‐West Diabetes Foot Care Study: Incidence of, and Risk Factors For, New Diabetic Foot Ulceration in a Community‐Based Patient Cohort,” Diabetic Medicine 19, no. 5 (May 2002): 377–384, 10.1046/j.1464-5491.2002.00698.x. 12027925

[48] S. A. Bus , I. C. N. Sacco , M. Monteiro‐Soares , et al., “Guidelines on the Prevention of Foot Ulcers in Persons With Diabetes (IWGDF 2023 Update),” Diabetes/Metabolism Research and Reviews 40, no. 3 (March 2024): e3651, 10.1002/dmrr.3651. 37302121

[49] R. S. Hernandez , R. Z. Gonzalez‐Marino , L. R. O. Jimenez , and B. D. Gurpide , “Prevalence of Amputation in HD Patients: A dialysis Program Without Socks. 60th ERA Congress. Virtual,” supplement, Nephrology Dialysis Transplantation 38, no. s1 (2023): i1217, 10.1093/ndt/gfad063d_4430.

[50] V. A. Ozdemir and N. Nural , “Risk Factors and Frequency of Foot Ulceration in Patients Receiving Chronic Hemodialysis Treatment,” Advances in skin & wound care 37, no. 4 (2024): 203–210, 10.1097/asw.0000000000000117. 38506581

[51] N. J. Jones , J. Chess , S. Cawley , A. O. Phillips , and S. G. Riley , “Prevalence of Risk Factors for Foot Ulceration in a General Haemodialysis Population,” International Wound Journal 10, no. 6 (December 2013): 683–688, 10.1111/j.1742-481X.2012.01044.x. 22891957 PMC7950860

[52] S. Morbach , H. Furchert , U. Gröblinghoff , et al., “Long‐Term Prognosis of Diabetic Foot Patients and Their Limbs: Amputation and Death Over the Course of a Decade,” Diabetes Care 35, no. 10 (2012): 2021–2027, 10.2337/dc12-0200. 22815299 PMC3447849

[53] K. Ogurtsova , S. Morbach , B. Haastert , et al., “Cumulative Long‐Term Recurrence of Diabetic Foot Ulcers in Two Cohorts From Centres in Germany and the Czech Republic,” Diabetes Research and Clinical Practice 172 (2021): 108621, 10.1016/j.diabres.2020.108621. 33316312

[54] NHS Digital , “National Diabetes Foot Care Audit 2020 to 2025,”November 2025, https://digital.nhs.uk/data‐and‐information/publications/statistical/national‐diabetes‐footcare‐audit/2025.

[55] P. Wijewickrama , M. Onyema , H. Eid , et al., “Standards of Diabetes Care and Burden of Hypoglycaemia in People With Diabetes on Peritoneal Dialysis: Results From a Real‐World Clinical Audit,” Peritoneal Dialysis International 44, no. 3 (2024): 216–220, 10.1177/08968608231195492. 37702352

[56] J. H. Seo , H. S. Lee , and Y. R. Choi , “Perioperative Risk Factors for Early Major Amputation Following First‐Time Diabetic Forefoot Amputation,” Foot & Ankle International 45, no. 10 (2024): 1111–1121, 10.1177/10711007241262792. 39075755

[57] P. Chen , N. C. Vilorio , K. Dhatariya , et al., “Effectiveness of Interventions to Enhance Healing of Chronic Foot Ulcers in Diabetes: A Systematic Review,” Diabetes 40, no. 3 (March 2024): e3786, 10.1002/dmrr.3786. 38507616

[58] C. McIntosh and C. MacGilchrist , “The Association Between Declining Kidney Function and Diabetic Foot Disease,” Diabetic Foot Journal 21, no. 2 (2018): 96–99.

[59] P. Song , D. Rudan , Y. Zhu , et al., “Global, Regional, and National Prevalence and Risk Factors for Peripheral Artery Disease in 2015: An Updated Systematic Review and Analysis,” Lancet Global Health 7, no. 8 (August 2019): e1020–e1030, 10.1016/s2214-109x(19)30255-4. 31303293

[60] C. L. B. Ho , H. J. Chih , P. S. Garimella , K. Matsushita , S. Jansen , and C. M. Reid , “Prevalence and Risk Factors of Peripheral Artery Disease in a Population With Chronic Kidney Disease in Australia: A Systematic Review and meta‐analysis,” Nephrology 26, no. 10 (October 2021): 798–808, 10.1111/nep.13914. 34156137

[61] M. Kallio , C. Forsblom , P. H. Groop , L. Groop , and M. Lepäntalo , “Development of New Peripheral Arterial Occlusive Disease in Patients With Type 2 Diabetes During a Mean follow‐up of 11 Years,” Diabetes Care 26, no. 4 (April 2003): 1241–1245, 10.2337/diacare.26.4.1241. 12663604

[62] P. Lanzer , M. Boehm , V. Sorribas , et al., “Medial Vascular Calcification Revisited: Review and Perspectives,” European Heart Journal 35, no. 23 (June 2014): 1515–1525, 10.1093/eurheartj/ehu163. 24740885 PMC4072893

[63] H. K. Aggarwal , S. Sood , D. Jain , V. Kaverappa , and S. Yadav , “Evaluation of Spectrum of Peripheral Neuropathy in Predialysis Patients With Chronic Kidney Disease,” Renal Failure 35, no. 10 (2013): 1323–1329, 10.3109/0886022x.2013.828261. 23964701

[64] D. B. Jasti , S. Mallipeddi , A. Apparao , B. Vengamma , V. Sivakumar , and S. Kolli , “A Clinical and Electrophysiological Study of Peripheral Neuropathies in Predialysis Chronic Kidney Disease Patients and Relation of Severity of Peripheral Neuropathy With Degree of Renal Failure,” Journal of Neurosciences in Rural Practice 8, no. 4 (October–December 2017): 516–524, 10.4103/jnrp.jnrp_186_17. 29204008 PMC5709871

[65] A. J. Boulton , L. Vileikyte , G. Ragnarson‐Tennvall , and J. Apelqvist , “The Global Burden of Diabetic Foot Disease,” Lancet 366, no. 9498 (November 2005): 1719–1724, 10.1016/s0140-6736(05)67698-2. 16291066

[66] A. Şeker , M. Usta , S. Gönüllü , et al., “Frailty and Peripheral Neuropathy in Hemodialysis Patients: Clinical and Electrophysiological Correlations,” Renal Failure 47, no. 1 (December 2025): 2547305, 10.1080/0886022x.2025.2547305. 40840867 PMC12372488

[67] L. A. Lavery , D. G. Armstrong , and A. J. Boulton , “Ankle Equinus Deformity and Its Relationship to High Plantar Pressure in a Large Population With Diabetes Mellitus,” Journal of the American Podiatric Medical Association 92, no. 9 (October 2002): 479–482, 10.7547/87507315-92-9-479. 12381796

[68] K. Ogurtsova , S. Morbach , B. Haastert , and A. Icks , “History of Diabetic Foot Ulcer Increases the Risk of Recurrence in a Long‐Term Follow‐Up Cohort in Germany. 56th Annual Meeting of the European Association for the Study of Diabetes, EASD 2020. Virtual,” Diabetologia 63, no. 1 (2020): S398–S399, 10.1016/j.diabres.2020.108621.

[69] A. Tarricone , T. L. Coye , A. Gee , B. Najafi , M. C. Siah , and L. A. Lavery , “The Dialysis Foot‐ the Impact of Presenting Estimated Glomerular Filtration Rate on Clinical Outcomes in Patients Hospitalized With Diabetic Foot Infections,” International Wound Journal 22, no. 5 (2025): e70122, 10.1111/iwj.70122. 40320291 PMC12050157

[70] T. S. Oh , H. S. Lee , and J. P. Hong , “Diabetic Foot Reconstruction Using Free Flaps Increases 5‐Year‐Survival Rate,” Journal of Plastic, Reconstructive and Aesthetic Surgery 66, no. 2 (2013): 243–250, 10.1016/j.bjps.2012.09.024. 23098584

[71] Y. Honda , K. Hirano , M. Yamawaki , et al., “Wound Healing of Critical Limb Ischemia With Tissue Loss in Patients on Hemodialysis,” Vascular 25, no. 3 (2017): 272–282, 10.1177/1708538116673015. 27758848

[72] A. Akturk , J. J. van Netten , M. Vermeer , et al., “Improved Outcomes in Patients With Diabetic Foot Ulcers Despite of Differences in Baseline Characteristics,” Wound Repair and Regeneration 29, no. 6 (2021): 912–919, 10.1111/wrr.12976. 34665904

[73] A. Hartemann‐Heurtier , G. Ha Van , J. P. Danan , et al., “Outcome of Severe Diabetic Foot Ulcers After Standardised Management in a Specialised Unit,” Diabetes & Metabolism 28, no. 6 Pt 1 (2002): 477–484, PMID: 12522328. 12522328

[74] G. Ha Van , C. Amouyal , O. Bourron , et al., “Diabetic Foot Ulcer Management in a Multidisciplinary Foot Centre: One‐Year Healing, Amputation and Mortality Rate,” Journal of Wound Care 29, no. 8 (2020): 464–471, PMID: 32804035, 10.12968/jowc.2020.29.8.464. 32804035

[75] C. Altobelli , F. C. Fabiani , P. Anastasio , et al., “Effects of Rheopheresis in Dialysis Patients With Peripheral Artery Disease and Diabetic Foot Ulcers: A Multicentric Italian study,” Journal of Clinical Apheresis 39, no. 4 (2024): e22132, 10.1002/jca.22132. 39105437

[76] M. Meloni , L. Giurato , V. Izzo , et al., “Long Term Outcomes of Diabetic Haemodialysis Patients With Critical Limb Ischemia and Foot Ulcer,” Diabetes Research and Clinical Practice 116 (June 2016): 117–122, 10.1016/j.diabres.2016.04.030. 27321326

[77] G. Messenger , R. Masoetsa , I. Hussain , S. Devarajan , and M. Jahromi , “Diabetic Foot Ulcer Outcomes From a Podiatry Led Tertiary Service in Kuwait,” Diabetic Foot and Ankle 9, no. 1 (2018): 1471927, 10.1080/2000625x.2018.1471927. 29868165 PMC5974709

[78] D. Q. Thai , Y. K. Jung , H. M. Hahn , and I. J. Lee , “Factors Affecting the Outcome of Lower Extremity Osteomyelitis Treated With Microvascular Free Flaps: An Analysis of 65 Patients,” Journal of Orthopaedic Surgery and Research 16, no. 1 (2021): 535, 10.1186/s13018-021-02686-x. 34452615 PMC8393737

[79] M. Utsunomiya , M. T. Tomita , M. K. Kinoshita , N. Ohura , and M. Nakamura , “Prognostic Factor of Diabetic and Ischemic Foot Ulcer, Multi‐Center, Multi Department Observational Study. European Society of Cardiology, ESC Congress 2017. Barcelona Spain,” supplement, European Heart Journal 38, no. s1 (2017): 280–281, 10.1093/eurheartj/ehx502.P1408.28182231

[80] G. Ha Van , C. Amouyal , C. Aubert , et al., “Treatment of a New Diabetic Foot Ulcer in a Diabetic Foot Service: A One Year Follow Up Prospective Study of 347 Patients,” supplement, Diabetologia 60, no. s1 (2017): S465, 10.1007/s00125-017-4350-z.

[81] C. D. Owens , K. J. Ho , S. Kim , et al., “Refinement of Survival Prediction in Patients Undergoing Lower Extremity Bypass Surgery: Stratification by Chronic Kidney Disease Classification,” Journal of Vascular Surgery 45, no. 5 (May 2007): 944–952, 10.1016/j.jvs.2007.01.025. 17391900

[82] M. Meloni , V. Izzo , L. Giurato , et al., “Recurrence of Critical Limb Ischemia After Endovascular Intervention in Patients With Diabetic Foot Ulcers,” Advances in Wound Care 7, no. 6 (2018): 171–176, 10.1089/wound.2017.0778. 29892493 PMC5994148

[83] H. Yasuhara , S. Naka , H. Yanagie , and H. Nagawa , “Influence of Diabetes on Persistent Nonhealing Ischemic Foot Ulcer in End‐Stage Renal Disease,” World Journal of Surgery 26, no. 11 (2002): 1360–1364, 10.1007/s00268-002-6335-3. 12447564

[84] M. Lepantalo , L. Fiengo , and F. Biancari , “Peripheral Arterial Disease in Diabetic Patients With Renal Insufficiency: A Review,” supplement, Diabetes 28, no. s1 (2012): 40–45, 10.1002/dmrr.2233. 22271722

[85] M. Albers , M. Romiti , N. De Luccia , F. C. Brochado‐Neto , I. Nishimoto , and C. A. B. Pereira , “An Updated Meta‐Analysis of Infrainguinal Arterial Reconstruction in Patients With End‐Stage Renal Disease,” Journal of Vascular Surgery 45, no. 3 (2007): 536–542, 10.1016/j.jvs.2006.11.036. 17257801

[86] M. C. Benjamin , K. W. Dane , and S. Senthil , “Postoperative Outcomes Among Dialysis Patients Undergoing Hip Fracture Repair,” Geriatric Orthopaedic Surgery & Rehabilitation (2023), 10.1177/21514593231195992. PMC10437044 37600450

[87] M. Plantz , R. Bergman , E. Gerlach , M. Mutawakkil , M. Patel , and A. Kadakia , “Comparing Perioperative Outcomes After Transmetatarsal Amputation in Patients With or Without Peripheral Vascular Disease,” Journal of Foot and Ankle Research 18, no. 1 (2025): e70026, 10.1002/jfa2.70026. 39924627 PMC11807761

[88] D. Nandakumar , M. J. Johnson , L. A. Lavery , et al., “Lower Extremity Amputation Rates in Patients With Chronic Kidney Disease: A Database Study Comparing Patients With and Without Diabetes Mellitus,” Journal of Diabetic Complications 38, no. 11 (November 2024): 108876, 10.1016/j.jdiacomp.2024.108876. 39378758

[89] W. A. Townley , T. W. Carrell , M. P. Jenkins , J. H. Wolfe , and N. J. Cheshire , “Critical Limb Ischemia in the Dialysis‐Dependent Patient: Infrainguinal Vein Bypass Is Justified,” Vascular and Endovascular Surgery 40, no. 5 (October–November 2006): 362–366, 10.1177/1538574406293739. 17038569

[90] B. Ramanan , H. Jeon‐Slaughter , X. Chen , J. G. Modrall , and S. Tsai , “Comparison of Open and Endovascular Procedures in Patients With Critical Limb Ischemia on Dialysis,” Journal of Vascular Surgery 70, no. 4 (2019): 1217–1224, 10.1016/j.jvs.2018.12.054. 30922740

[91] A. Kodama , M. Sugimoto , S. Kuma , J. Okazaki , S. Mii , and K. Komori , “Clinical Outcomes After Infrainguinal Bypass Grafting for Critical Limb Ischaemia in Patients With Dialysis‐Dependent End‐Stage Renal Failure,” European Journal of Vascular and Endovascular Surgery 48, no. 6 (December 2014): 695–702, 10.1016/j.ejvs.2014.08.022. 25281532

[92] E. H. Weissler , D. I. Narcisse , J. A. Rymer , et al., “Characteristics and Outcomes of Patients With Diabetes Mellitus Undergoing Peripheral Vascular Intervention for Infrainguinal Symptomatic Peripheral Artery Disease,” Vascular and Endovascular Surgery 55, no. 2 (2021): 124–134, 10.1177/1538574420968671. 33094679 PMC8150867

[93] J. A. Rubio , S. Jimenez , and J. L. Lazaro‐Martinez , “Mortality in Patients With Diabetic Foot Ulcers: Causes, Risk Factors, and Their Association With Evolution and Severity of Ulcer,” Journal of Clinical Medicine 9, no. 9 (2020): 1–14, 10.3390/jcm9093009. PMC7565534 32961974

[94] M. Venermo , F. Biancari , E. Arvela , et al., “The Role of Chronic Kidney Disease as a Predictor of Outcome After Revascularisation of the Ulcerated Diabetic Foot,” Diabetologia 54, no. 12 (December 2011): 2971–2977, 10.1007/s00125-011-2279-1. 21845468

[95] P. W. Eggers , D. Gohdes , and J. Pugh , “Nontraumatic Lower Extremity Amputations in the Medicare End‐Stage Renal Disease Population,” Kidney Int 56, no. 4 (1999): 1524–1533, 10.1046/j.1523-1755.1999.00668.x. 10504504

[96] A. M. O'Hare , P. Bacchetti , M. Segal , C. Y. Hsu , and K. L. Johansen , and Dialysis Morbidity and Mortality Study Waves , “Factors Associated With Future Amputation Among Patients Undergoing Hemodialysis: Results From the Dialysis Morbidity and Mortality Study Waves 3 and 4,” American Journal of Kidney Diseases 41, no. 1 (2003): 162–170, 10.1053/ajkd.2003.50000. 12500233

[97] R. A. Speckman , D. L. Frankenfield , S. H. Roman , et al., “Diabetes is the Strongest Risk Factor for Lower‐Extremity Amputation in New Hemodialysis Patients,” Diabetes Care 27, no. 9 (2004): 2198–2203, 10.2337/diacare.27.9.2198. 15333484

[98] B. G. Jaar , B. C. Astor , J. S. Berns , and N. R. Powe , “Predictors of Amputation and Survival Following Lower Extremity Revascularization in Hemodialysis Patients,” Kidney International 65, no. 2 (2004): 613–620, 10.1111/j.1523-1755.2004.00420.x. 14717932

[99] M. G. Sheahan , A. D. Hamdan , J. R. Veraldi , et al., “Lower Extremity Minor Amputations: The Roles of Diabetes Mellitus and Timing of Revascularization,” Journal of Vascular Surgery 42, no. 3 (2009): 476–480, 10.1016/j.jvs.2005.05.003. 16171590

[100] A. Tarricone , A. Gee , A. J. Boulton , L. Rogers , and L. A. Lavery , “Uncontrolled Diabetes Is a Strong Predictor of Amputation in End Stage Renal Disease Patients on Hemodialysis,” Annals of Vascular Surgery 114 (May 2025): 313–319, 10.1016/j.avsg.2024.12.057. 39736381

[101] M. Meloni , V. Izzo , L. Giurato , J. L. Lázaro‐Martínez , and L. Uccioli , “Prevalence, Clinical Aspects and Outcomes in a Large Cohort of Persons With Diabetic Foot Disease: Comparison Between Neuropathic and Ischemic Ulcers,” J Clin Med 9, no. 6 (2020): 1780, 10.3390/jcm9061780. 32521700 PMC7356179

[102] M. Meloni , A. Andreadi , E. Bellizzi , et al., “A Multidisciplinary Team Reduces In‐Hospital Clinical Complications and Mortality in Patients With Diabetic Foot Ulcers,” Diabetes/Metabolism Research and Reviews 39, no. 7 (2023): e3690, 10.1002/dmrr.3690. 37422897

[103] M. Meloni , V. Izzo , V. Da Ros , et al., “Characteristics and Outcome for Persons With Diabetic Foot Ulcer and No‐Option Critical Limb Ischemia,” Journal of Clinical Medicine 9, no. 11 (2020): 3745, 10.3390/jcm9113745. 33233329 PMC7700155

[104] Y. Orimoto , T. Ohta , H. Ishibashi , et al., “The Prognosis of Patients on Hemodialysis With Foot Lesions,” Journal of Vascular Surgery 58, no. 5 (November 2013): 1291–1299, 10.1016/j.jvs.2013.05.027. 23810259

[105] S. Miyajima , A. Shirai , S. Yamamoto , N. Okada , and T. Matsushita , “Risk Factors for Major Limb Amputations in Diabetic Foot Gangrene Patients,” Diabetes Research and Clinical Practice 71, no. 3 (2006): 272–279, 10.1016/j.diabres.2005.07.005. 16139385

[106] R. B. Paisey , M. Waterson , J. Davis , et al., “Impaired But Improving Outcomes for Those With Diabetes Related Foot Ulceration and Renal Failure,” supplement, Diabetic Medicine 29, no. s1 (2012): 149, 10.1111/j.1464-5491.2011.03555_2.x.

[107] A. Ndip , A. Vardhan , K. Breislin , and A. J. Boulton , “High Mortality Rates From Foot Complications in Diabetic Patients on Dialysis,” supplement, Diabetes 61, no. s1 (2012): A32, 72nd Scientific Sessions of the American Diabetes Association, 10.2337/db12-1-377.

[108] D. W. Shim , W. Lee , K. H. Park , et al., “Risk Factors and Mortality for Amputations in the Diabetic Foot: A Nationwide Cohort Study,” Diabetes Research and Clinical Practice 234 (2026): 112435, 10.1016/j.diabres.2025.112435. 40854328

[109] S. Namgoong , S. Jung , S. Han , S. Jeong , E. Dhong , and W. Kim , “Risk Factors for Major Amputation in Hospitalised Diabetic Foot Patients,” supplement, International Wound Journal 13, no. s1 (2016): 13–19, 10.1111/iwj.12526. 26478562 PMC7949865

[110] E. Hamilton , W. Davis , M. Baba , and T. Davis , “Temporal Trends in Minor and Major Lower Extremity Amputation in People With Type 2 Diabetes: The Fremantle Diabetes Study,” Diabetes and Vascular Disease Research 20, no. 1 (2023): 14791641231154162, 10.1177/14791641231154162. 36715218 PMC9903017

[111] N. S. R. Lan , J. Hiew , I. Ferreira , et al., “The Combined Impact of Chronic Kidney Disease and Ulcer Severity on Incident Cardiovascular Events in Patients With Diabetes‐Related Foot Ulceration,” Physiological Reports 13, no. 11 (2025): e70415, 10.14814/phy2.70415. 40474776 PMC12141929

[112] L. Stuart , L. Kimmel , and A. Jolly , “Incidence of Lower Limb Amputation in Central Australia,” Australian Health Review: A Publication of the Australian Hospital Association 45, no. 3 (2021): 361–367, 10.1071/ah20182. 33647229

[113] G. Wolf , N. Muller , M. Busch , et al., “Diabetic Foot Syndrome and Renal Function in Type 1 and 2 Diabetes Mellitus Show Close Association,” Nephrol Dial Transplant 24, no. 6 (2009): 1896–1901, 10.1093/ndt/gfn724. 19131351

[114] Y. He , H. Qian , L. Xu , et al., “Association Between Estimated Glomerular Filtration Rate and Outcomes in Patients With Diabetic Foot Ulcers: A 3‐Year Follow‐Up Study,” European Journal of Endocrinology 177, no. 1 (2017): 41–50, 10.1530/eje-17-0070. 28424173

[115] J. Zhang , D. Chen , X. Li , et al., “The Association Between Estimated Glomerular Filtration Rate and Prognosis in Patients With Diabetic Foot Osteomyelitis,” International Wound Journal 19, no. 7 (2022): 1650–1657, 10.1111/iwj.13765. 35080116 PMC9615297

[116] L. Prompers , N. Schaper , J. Apelqvist , et al., “Prediction of Outcome in Individuals With Diabetic Foot Ulcers: Focus on the Differences Between Individuals With and Without Peripheral Arterial Disease. The EURODIALE Study,” Diabetologia 51, no. 5 (2008): 747–755, 10.1007/s00125-008-0940-0. 18297261 PMC2292424

[117] C. Randon , B. Jacobs , F. De Ryck , K. Van Landuyt , and F. Vermassen , “A 15‐Year Experience With Combined Vascular Reconstruction and Free Flap Transfer for Limb‐Salvage,” European Journal of Vascular and Endovascular Surgery 38, no. 3 (2009): 338–345, 10.1016/j.ejvs.2009.06.005. 19596597

[118] W. L. Tay , T. T. Chong , S. L. Chan , et al., “Two‐Year Clinical Outcomes Following Lower Limb Endovascular Revascularisation for Chronic Limb‐Threatening Ischaemia at a Tertiary Asian Vascular Centre in Singapore,” Singapore Medical Journal 63, no. 2 (2022): 79–85, 10.11622/smedj.2020104. 32668837 PMC9251224

[119] O. O. Adeleye , A. O. Williams , A. O. Dada , I. Okpe , I. Ezeani , and M. Enamino , “Predictors of Intra‐Hospital Mortality in Patients With Diabetic Foot Ulcers in Nigeria: Data From the MEDFUN study,” BMC Endocr Disord 20, no. 1 (2020): 134, 10.1186/s12902-020-00614-4. PMC7455894 32859203

[120] O. Akha , Z. Kashi , and A. Makhlough , “Correlation Between Amputation of Diabetic Foot and Nephropathy,” Iranian Journal of Kidney Diseases 4, no. 1 (2010): 27–31, PMID: 20081301. 20081301

[121] W. Alashek , C. McIntyre , and M. Taal , “Morbidity of Diabetic End‐Stage Kidney Disease Patients Treated by Dialysis in Libya,”supplement, Diabetes, Obesity and Metabolism 12, no. s1 (2010): 76–77, 10.1111/j.1463-1326.2010.01284.x.

[122] D. C. Jupiter , J. C. Thorud , C. J. Buckley , and N. Shibuya , “The Impact of Foot Ulceration and Amputation on Mortality in Diabetic Patients. I: From Ulceration to Death, a Systematic Review,” International Wound Journal 13, no. 5 (October 2016): 892–903, 10.1111/iwj.12404. 25601358 PMC7950078

[123] S. F. Shaw , J. J. Sim , H. Zhou , J. Shi , and S. J. Jacobsen , “A Comparison of Death Records Between the United States Renal Data System and a Large Integrated Health Care System,” Kidney International Reports 5, no. 6 (June 2020): 912–915, 10.1016/j.ekir.2020.03.019. 32518873 PMC7270980

[124] D. K. Wukich , K. M. Raspovic , D. C. Jupiter , et al., “Amputation and Infection are the Greatest Fears in Patients With Diabetes Foot Complications,” Journal of Diabetes and its Complications 36, no. 7 (2022): 108222, 10.1016/j.jdiacomp.2022.108222. 35717355

[125] S. Gharibzadeh , J. Lee , P. Highton , et al., “Risk Factors for Development of Diabetic Foot Ulcer Disease in Two Large Contemporary UK Cohorts,” Diabetes, Obesity and Metabolism 27, no. 9 (June 2025): 4782–4792, 10.1111/dom.16519. PMC12326939 40555701

[126] M. B. Brennan , T. M. Hess , B. Bartle , et al., “Diabetic Foot Ulcer Severity Predicts Mortality Among Veterans With Type 2 Diabetes,” Journal of Diabetic Complications 31, no. 3 (March 2017): 556–561, 10.1016/j.jdiacomp.2016.11.020. PMC5328848 27993523

[127] N. V. Arinze , A. Gregory , J. M. Francis , A. Farber , and V. C. Chitalia , “Unique Aspects of Peripheral Artery Disease in Patients With Chronic Kidney Disease,” Vascular Medicine 24, no. 3 (June 2019): 251–260, 10.1177/1358863x18824654. 30823859

[128] D. G. Armstrong , M. A. Swerdlow , A. A. Armstrong , M. S. Conte , W. V. Padula , and S. A. Bus , “Five Year Mortality and Direct Costs of Care for People With Diabetic Foot Complications are Comparable to Cancer,” Journal of Foot and Ankle Research 13, no. 1 (2020/03/24 2020): 16, 10.1186/s13047-020-00383-2. PMC7092527 32209136

[129] F. Serizawa , S. Sasaki , S. Fujishima , D. Akamatsu , H. Goto , and N. Amada , “Mortality Rates and Walking Ability Transition After Lower Limb Major Amputation in Hemodialysis Patients,” Journal of Vascular Surgery 64, no. 4 (October 2016): 1018–1025, 10.1016/j.jvs.2016.03.452. 27189770

[130] NHS England , National Diabetes Foot Care Audit 2018–2023, (2024), https://digital.nhs.uk/data‐and‐information/publications/statistical/national‐diabetes‐footcare‐audit/2018‐2023.

[131] N. Abi , A. Rossi , S. O. Pastan , R. E. Patzer , and J. L. Harding , “Sex and Cause‐specific Mortality Among US Adults Receiving Maintenance Dialysis: National US Cohort Study 2000–2021,” Kidney 6, no. 7 (February 2025): 1116–1126, 10.34067/kid.0000000741. PMC12338354 39951343

[132] K. L. Johansen , D. T. Gilbertson , S. Li , et al., “US Renal Data System 2023 Annual Data Report: Epidemiology of Kidney Disease in the United States,” American Journal of Kidney Diseases 83, no. 4 S1 (April 2024): A8–a13, 10.1053/j.ajkd.2024.01.001. 38519262

[133] W. H. Lim , J. H. C. Chen , K. Minas , et al., “Sex Disparity in Cause‐Specific and All‐Cause Mortality Among Incident Dialysis Patients,” American Journal of Kidney Diseases 81, no. 2 (February 2023): 156–167.e1, 10.1053/j.ajkd.2022.07.007. 36029966

[134] C. B. Bowling , R. Zhang , H. Franch , et al., “Underreporting of Nursing Home Utilization on the CMS‐2728 in Older Incident Dialysis Patients and Implications for Assessing Mortality Risk,” BMC Nephrology 16, no. 1 (March 2015): 32, 10.1186/s12882-015-0021-9. 25880589 PMC4408561

[135] M. Vitale , E. Orsi , A. Solini , et al., “Independent Association of History of Diabetic Foot With All‐Cause Mortality in Patients With Type 2 Diabetes: The Renal Insufficiency and Cardiovascular Events (RIACE) Italian Multicenter Study,” Cardiovascular Diabetology 23, no. 1 (January 2024): 34, 10.1186/s12933-023-02107-9. 38218843 PMC10787405

[136] D. Martins‐Mendes , M. Monteiro‐Soares , E. J. Boyko , et al., “The Independent Contribution of Diabetic Foot Ulcer on Lower Extremity Amputation and Mortality Risk,” Journal of Diabetic Complications 28, no. 5 (September–October 2014): 632–638, 10.1016/j.jdiacomp.2014.04.011. PMC4240944 24877985

[137] L. Chen , S. Sun , Y. Gao , and X. Ran , “Global Mortality of Diabetic Foot Ulcer: A Systematic Review and Meta‐Analysis of Observational Studies,” Diabetes, Obesity and Metabolism 25, no. 1 (January 2023): 36–45, 10.1111/dom.14840. 36054820

[138] D. K. Wukich , K. M. Raspovic , and N. C. Suder , “Patients With Diabetic Foot Disease Fear Major Lower‐Extremity Amputation More Than Death,” Foot & Ankle Specialist 11, no. 1 (February 2018): 17–21, 10.1177/1938640017694722. 28817962

[139] M. R. Kaminski , A. Raspovic , L. P. McMahon , et al., “Risk Factors for Foot Ulceration and Lower Extremity Amputation in Adults With End‐Stage Renal Disease on Dialysis: A Systematic Review and Meta‐Analysis,” Nephrology Dialysis Transplantation 30, no. 10 (October 2015): 1747–1766, 10.1093/ndt/gfv114. 25943598

[140] M. R. Kaminski , A. Raspovic , L. P. McMahon , et al., “Factors Associated With Foot Ulceration and Amputation in Adults on Dialysis: A Cross‐Sectional Observational Study,” BMC Nephrology 18, no. 1 (September 2017): 293, 10.1186/s12882-017-0711-6. 28886703 PMC5591526

[141] C. A. Abbott , L. Vileikyte , S. Williamson , A. L. Carrington , and A. J. Boulton , “Multicenter Study of the Incidence of and Predictive Risk Factors for Diabetic Neuropathic Foot Ulceration,” Diabetes Care 21, no. 7 (July 1998): 1071–1075, 10.2337/diacare.21.7.1071. 9653597

[142] C. A. Abbott , A. P. Garrow , A. L. Carrington , J. Morris , E. R. Van Ross , and A. J. Boulton , “Foot Ulcer Risk Is Lower in South‐Asian and African‐Caribbean Compared With European Diabetic Patients in the U.K.: The North‐West Diabetes Foot Care Study,” Diabetes Care 28, no. 8 (August 2005): 1869–1875, 10.2337/diacare.28.8.1869. 16043725

[143] K. Snow , R. Patzer , S. Patel , and J. Harding , “County‐Level Characteristics Associated With Variation in ESKD Mortality in the United States, 2010–2018,” Kidney 3, no. 5 (2022): 891–899, 10.34067/KID.0007872021. PMC9438422 36128479

[144] W. Marshall , “Don't Get Cold Feet—A Vital Screening Opportunity for Diabetic Haemodialysis Patients,” Clinical Medicine, Journal of the Royal College of Physicians of London 24 (2024): 100138, 10.1016/j.clinme.2024.100138.

[145] W. Jeffcoate , F. Game , S. Morbach , M. Narres , K. Van Acker , and A. Icks , “Assessing Data on the Incidence of Lower Limb Amputation in Diabetes,” Diabetologia 6 (2021): 1442–1446, 10.1007/s00125-021-05440-4. 33783587

[146] N. M. McGrath and B. A. Curran , “Recent Commencement of Dialysis Is a Risk Factor for Lower‐Extremity Amputation in a High‐Risk Diabetic Population,” Diabetes Care 23, no. 3 (March 2000): 432–433, 10.2337/diacare.23.3.432. 10868890

[147] J. Lipscombe , S. V. Jassal , S. Bailey , J. M. Bargman , S. Vas , and D. G. Oreopoulos , “Chiropody May Prevent Amputations in Diabetic Patients on Peritoneal Dialysis,” Peritoneal Dialysis International 23, no. 3 (May–June 2003): 255–259, 10.1177/089686080302300307. 12938826

[148] C. Young and A. K. Myers , “Racial and Ethnic Disparities in Diabetes Clinical Care and Management: A Narrative Review,” Endocrine Practice 29, no. 4 (2023): 295–300, 10.1016/j.eprac.2022.11.013. 36464131

[149] H. Yasuhara , T. Hattori , and O. Shigeta , “Significance of Phlebosclerosis in Non‐Healing Ischaemic Foot Ulcers of End‐Stage Renal Disease,” European Journal of Vascular and Endovascular Surgery 36, no. 3 (September 2008): 346–352, 10.1016/j.ejvs.2008.05.003. 18586533

[150] A. H. Frankel , M. Wahba , V. Ashworth , et al., “Management of Adults With Diabetes on Dialysis: Summary of Recommendations of the Joint British Diabetes Societies Guidelines 2022,” Diabetic Medicine 40, no. 4 (April 2023): e15027, 10.1111/dme.15027. 36524709

[151] D. J. Margolis , N. Mitra , D. S. Malay , et al., “Further Evidence That Wound Size and Duration Are Strong Prognostic Markers of Diabetic Foot Ulcer Healing,” Wound Repair and Regeneration 30, no. 4 (2022): 487–490, 10.1111/wrr.13019. 35470507 PMC9246994

[152] J. W. Walsh , O. J. Hoffstad , M. O. Sullivan , and D. J. Margolis , “Association of Diabetic Foot Ulcer and Death in a Population‐Based Cohort From the United Kingdom,” Diabetic Medicine 33, no. 11 (November 2016): 1493–1498, 10.1111/dme.13054. 26666583

[153] R. J. Hinchliffe , B. Kirk , D. Bhattacharjee , S. Roe , W. Jeffcoate , and F. Game , “The Effect of Haemodialysis on Transcutaneous Oxygen Tension in Patients With Diabetes‐A Pilot Study,” Nephrology Dialysis Transplantation 21, no. 7 (July 2006): 1981–1983, 10.1093/ndt/gfl241. 16702198

[154] É Senneville , B. A. Lipsky , Z. G. Abbas , et al., “Diagnosis of Infection in the Foot in Diabetes: A Systematic Review,” supplement, Diabetes/Metabolism Research and Reviews 36, no. s1 (March 2020): e3281, 10.1002/dmrr.3281. 32176440

[155] R. J. Hinchliffe , W. J. Jeffcoate , and F. L. Game , “Diabetes, Established Renal Failure and the Risk to the Lower Limb,” Practical Diabetes International 23, no. 1 (2006): 28–32, 10.1002/pdi.886.

[156] M. Monteiro‐Soares , E. J. Hamilton , D. A. Russell , et al., “Guidelines on the Classification of Foot Ulcers in People With Diabetes (IWGDF 2023 Update),” Diabetes/Metabolism Research and Reviews 40, no. 3 (March 2024): e3648, 10.1002/dmrr.3648. 37179483

